# Assessing the relative efficacy of interleukin-17 and interleukin-23 targeted treatments for moderate-to-severe plaque psoriasis: A systematic review and network meta-analysis of PASI response

**DOI:** 10.1371/journal.pone.0220868

**Published:** 2019-08-14

**Authors:** Laura M. Sawyer, Kinga Malottki, Celia Sabry-Grant, Najeeda Yasmeen, Emily Wright, Anne Sohrt, Emma Borg, Richard B. Warren

**Affiliations:** 1 Symmetron Limited, London, England, United Kingdom; 2 LEO Pharma A/S, Ballerup, Denmark; 3 Dermatology Centre, Salford Royal NHS Foundation Trust, Manchester NIHR Biomedical Research Centre, The University of Manchester, Manchester, England, United Kingdom; University of Mississippi Medical Center, UNITED STATES

## Abstract

**Introduction:**

New generation biologics, including interleukin (IL)-17 and IL-23 inhibitors, have delivered higher rates of skin clearance than older treatments in head-to-head studies. However, studies comparing these new biologics directly to one another are limited.

**Objectives:**

To compare the short-term efficacy of available (or imminently available) biologic and non-biologic systemic therapies for treating patients with moderate-to-severe plaque psoriasis.

**Methods:**

A systematic review was undertaken to identify randomised controlled trials evaluating biologic treatments, apremilast and dimethyl fumarate. MEDLINE, MEDLINE In-Process, Embase and the Cochrane Library were searched from the 1st January 2000 to 22nd November 2018. A Bayesian network meta-analysis (NMA) using a random-effects multinomial likelihood model with probit link and meta-regression to adjust for cross-trial variation in placebo responses compared the efficacy of interventions at inducing different levels of Psoriasis Area and Severity Index (PASI) response during the induction period. A range of sensitivity analyses was undertaken.

**Results:**

Seventy-seven trials (34,816 patients) were included in the NMA. The base-case analysis showed that all active treatments were superior to placebo. IL-17 inhibitors, guselkumab and risankizumab were found to be more efficacious than tildrakizumab, ustekinumab, all TNF inhibitors and non-biologic systemic treatments at inducing all levels of PASI response. In addition, brodalumab, ixekizumab and risankizumab were significantly more efficacious than secukinumab; no significant difference was found in the comparison with guselkumab. The greatest benefit of brodalumab, ixekizumab, guselkumab, and risankizumab was seen for PASI 90 and PASI 100 response. Results were consistent across all analyses.

**Conclusions:**

In the NMA brodalumab, ixekizumab, risankizumab and guselkumab showed the highest levels of short-term efficacy. There were differences in efficacy between treatments within the same class. Longer-term analyses are needed to understand differences between these drugs beyond induction in what is a life-long condition.

## Introduction

Psoriasis is a chronic, immune‐mediated skin disease which affects approximately 100 million people worldwide [1]. It is estimated that in most developed countries between 1.5 and 5% of the population suffer from psoriasis, with the incidence highest in people in their twenties, thirties and sixties [2]. It affects men and women of all ages, although it is rare in children. The most common type is plaque psoriasis (*psoriasis vulgaris*), characterized by patches of erythema covered in plaques that can range in colour from silvery-white to yellow [3]. Approximately 20% of patients have moderate-to-severe disease [4, 5]. Psoriasis may affect patient’s self-esteem and lead to low quality of life, comparable to that of patients with heart disease or diabetes [6–10]. Many factors must be considered when selecting the best treatment for patients with plaque psoriasis.

Biologic therapies are generally licensed for treatment of patients eligible for systemic therapy; however, in practice they may be considered after other therapies have failed. For example, in the UK initial management of such patients may involve conventional systemic therapies such as methotrexate, cyclosporine, or acitretin [11]. Only patients with moderate-to-severe plaque psoriasis who do not respond to, or are intolerant of conventional systemic treatments are offered biologic systemic therapies, the phosphodiesterase 4 (PDE4) inhibitor apremilast or dimethyl fumarate (DMF) [4, 12–14]. In other countries, modern biologics may be prescribed earlier in the treatment pathway.

The first biologics used in psoriasis were anti-tumour necrosis factor (TNF) therapies, adalimumab, etanercept and infliximab [15]. Although their introduction transformed the treatment of the disease [16], in the majority of patients they do not provide complete skin clearance [17]. In addition, it is common for patients to discontinue anti-TNF treatment due to loss of response and adverse effects [18–20]. In 2009, the biologic therapy ustekinumab, which targets the interleukin (IL)-12/23 pathway, became available [21].

More recently, biologics targeting the IL-17 pathway have been introduced into clinical practice. The three approved IL-17 pathway inhibitors are secukinumab and ixekizumab, both IL-17A inhibitors, and brodalumab, which targets the IL-17-receptor A (IL-17RA) [22]. These treatments have been shown to be efficacious and safe and offer higher rates of complete or almost complete clearance compared to TNF inhibitors and ustekinumab [23–25]. Another class of systemic drugs potentially offering similarly high complete clearance rates target IL-23. These include guselkumab, tildrakizumab, and risankizumab [26–29]. Although data from clinical trials has shown these treatments to be more efficacious than anti-TNFs and ustekinumab [30–36], clinical trial data on their benefit compared to the more established IL-17 inhibitors is very limited [37, 38]. Rapid developments in the field of psoriasis treatments necessitate assessment of the efficacy of new drugs relative to older treatments and different drug classes.

The aim of this systematic review (SR) and network meta-analysis (NMA) was therefore, to compare the efficacy of IL-17 targeted drugs (brodalumab, ixekizumab, and secukinumab) to new IL-23 targeted treatments (guselkumab, risankizumab, and tildrakizumab), as well as other systemic biologic and non-biologic therapies using the latest evidence. Psoriasis Area and Severity Index (PASI) response was chosen as the measure of efficacy, as it captures both the severity and extent of psoriatic lesions. The PASI score is measured on a scale from 0 to 72 and response to treatment is often measured in terms of percentage decrease in the PASI score, with values of 50%, 75%, 90% and 100% usually reported in trials [39, 40].

## Materials and methods

The SR was carried out in accordance with a protocol developed prior to commencement of the review (details reported in S1 Text).

### Search strategy

Electronic databases (MEDLINE, MEDLINE In-Process, Embase and the Cochrane Library) were searched from the 1st January 2000 to 22nd November 2018 for articles published in English. Search terms included index and text terms for psoriasis, relevant interventions and study design. The full search strategies can be found in S1 Table. In addition, reference lists of included studies and any relevant systematic reviews or NMAs identified during title and abstract screening were scanned to identify further studies. Disease-specific and health economics and outcomes research conferences were also searched.

### Study selection

All articles identified in the electronic searches were added to an EndNote database and duplicates removed. Titles, abstracts and potentially relevant full texts of articles were assessed for inclusion by one reviewer, with another reviewer performing a 50% check at both stages. Discrepancies were resolved by discussion or, if necessary, by a third reviewer.

Randomised controlled trials (RCTs) in adult patients with moderate-to-severe chronic plaque psoriasis were included in the review. Interventions of interest included licensed doses of IL-17 inhibitors (brodalumab, secukinumab and ixekizumab), IL-23 inhibitors (tildrakizumab, guselkumab and risankizumab), IL-12/23 inhibitor ustekinumab, anti-TNF therapies (infliximab, etanercept, certolizumab pegol and adalimumab), PDE4 inhibitor apremilast and fumaric acid ester (FAE) dimethyl fumarate (DMF). Comparators included all interventions of interest, as well as placebo and unlicensed doses of biological and non-biological therapies. Biosimilar versions of drugs were considered as part of the evidence base for the originator drug. The outcome of interest was the proportion of patients achieving 50, 75, 90 and 100% improvement in their PASI score at the end of the induction period. Only articles published in English were considered eligible. A full set of inclusion and exclusion criteria can be found in S2 Table.

### Data extraction and quality assessment

Details of the study design, baseline patient characteristics, interventions, outcomes, timepoints and results were extracted by one reviewer and checked by another. Additional data from study investigators were not sought. The methodological quality of included studies was assessed by one independent reviewer and checked by another using the concise critical appraisal checklists in the National Institute for Health and Care Excellence (NICE) Single Technology Appraisal user guide [41].

### Network meta-analysis of PASI response

A hierarchical Bayesian NMA was used to combine results of all relevant studies allowing for all data from direct and indirect comparisons to be synthesised into a single set of effect sizes. The methods followed the recommended best practice for evidence synthesis [42].

The outcome of interest was the proportion of patients achieving each level of PASI response (PASI 50, PASI 75, PASI 90 and PASI 100) at the end of the induction phase (between 10 and 16 weeks). Data were analysed using an ordered probit model designed to estimate probabilities of achieving each level of PASI response [42]. PASI 50 responses in placebo arms of the included studies were utilised to inform the baseline event rates [43]. PASI response was modelled as a discrete dependent variable that takes ordered multinomial outcomes (PASI 50, PASI 75, PASI 90 and PASI 100). Cross-trial variation in placebo response rates has been observed in psoriasis [44] and is an indicator of between-study heterogeneity, which can introduce bias to synthesised comparisons of treatment outcomes [45–47]. Randomised trials in moderate-to-severe psoriasis vary by design, eligibility criteria, time to primary endpoint and concomitant emollient use, all of which could contribute to differences in placebo responses and consequently to relative effect sizes. Adjusting for differences in the placebo arms may account for heterogeneity between trials and could improve the model fit [42, 48–50]. This approach has previously been used in other NMAs comparing the efficacy of biological treatments for moderate-to-severe psoriasis [38, 51] and has been recommended by NICE in recognition of the variability in placebo response across trials [52]. Analyses adjusting and not adjusting for cross-trial variation in placebo-arm responses were carried out using random-effects assumptions and assessed for their goodness of fit. In accordance with NMA guidelines [49], fit was informed by the statistical significance of the regression coefficient and whether there was a reduction in between-trial heterogeneity. The model with the best fit was used to draw conclusions. Inconsistency between the direct and indirect evidence was assessed using a random-effects unrelated mean effects model [53]. Full details of the model used are shown in S2 Text.

WinBUGS version 1.4 [54] was used to perform all statistical analyses, using non-informative priors. After an initial burn-in of at least 20,000 simulations, convergence was confirmed through visual inspection the Brook-Gelman-Rubin diagnostic and history plots. Sampled parameters were then estimated using 20,000 simulations on three chains. Results were calculated as the absolute probabilities of response for each treatment and as risk ratios (RRs) for every pairwise comparison at each level of PASI response. Point estimates reflecting the median value are presented, along with 95% credible intervals (95% CrI), reflecting the range of true effects with 95% probability. Sensitivity analyses were performed to test the impact of excluding trials reporting <5% biologic exposed patients, using a different timepoint (12 weeks rather than 16 weeks) for secukinumab, and excluding studies with fewer than 50 patients per arm. PASI data inputs are reported in S3 Table.

## Results

### Identification of trials

**Fig 1:**
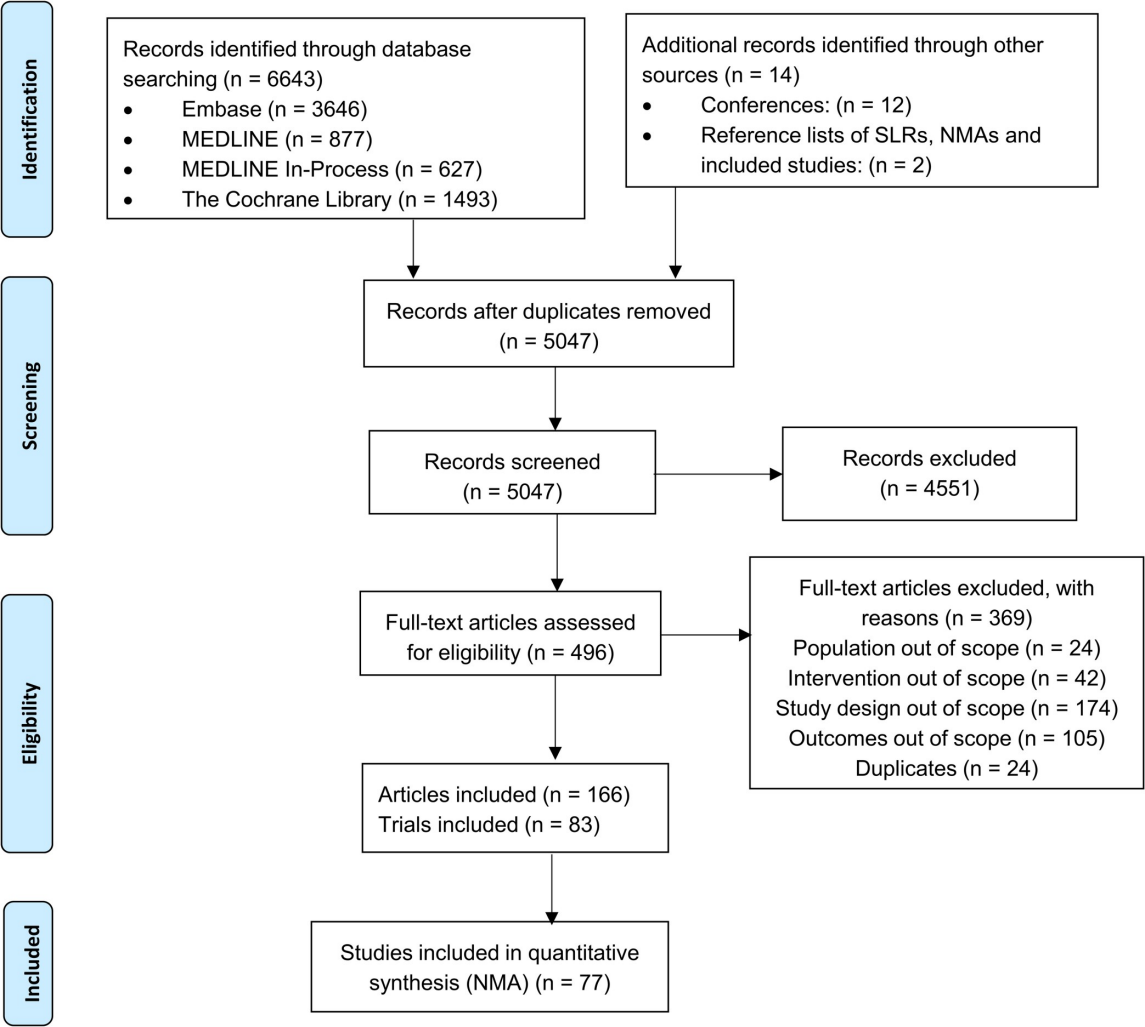
PRISMA flow diagram.

As shown in Fig 1, electronic database searches identified 6643 articles. A further fourteen were identified through searching conferences and reference lists of included studies, SRs and NMAs. After the removal of duplicates, 5047 titles and abstracts were screened, and subsequently 496 full texts were assessed for eligibility. Three-hundred and sixty-nine full text articles were excluded based on the population (n = 24), intervention (n = 42), study design (n = 174), outcomes (n = 105) and as duplicates (n = 24). References of excluded studies can be found in S4 Table. Eighty-three RCTs (reported in 127 articles) were included in the SR and 77 (including 34,816 patients) in the NMA. The trials not included in the NMA did not provide results at all [55–57] or in an appropriate format [58], or included a comparator that would not connect relevant interventions in the network [59, 60].

**Fig 2:**
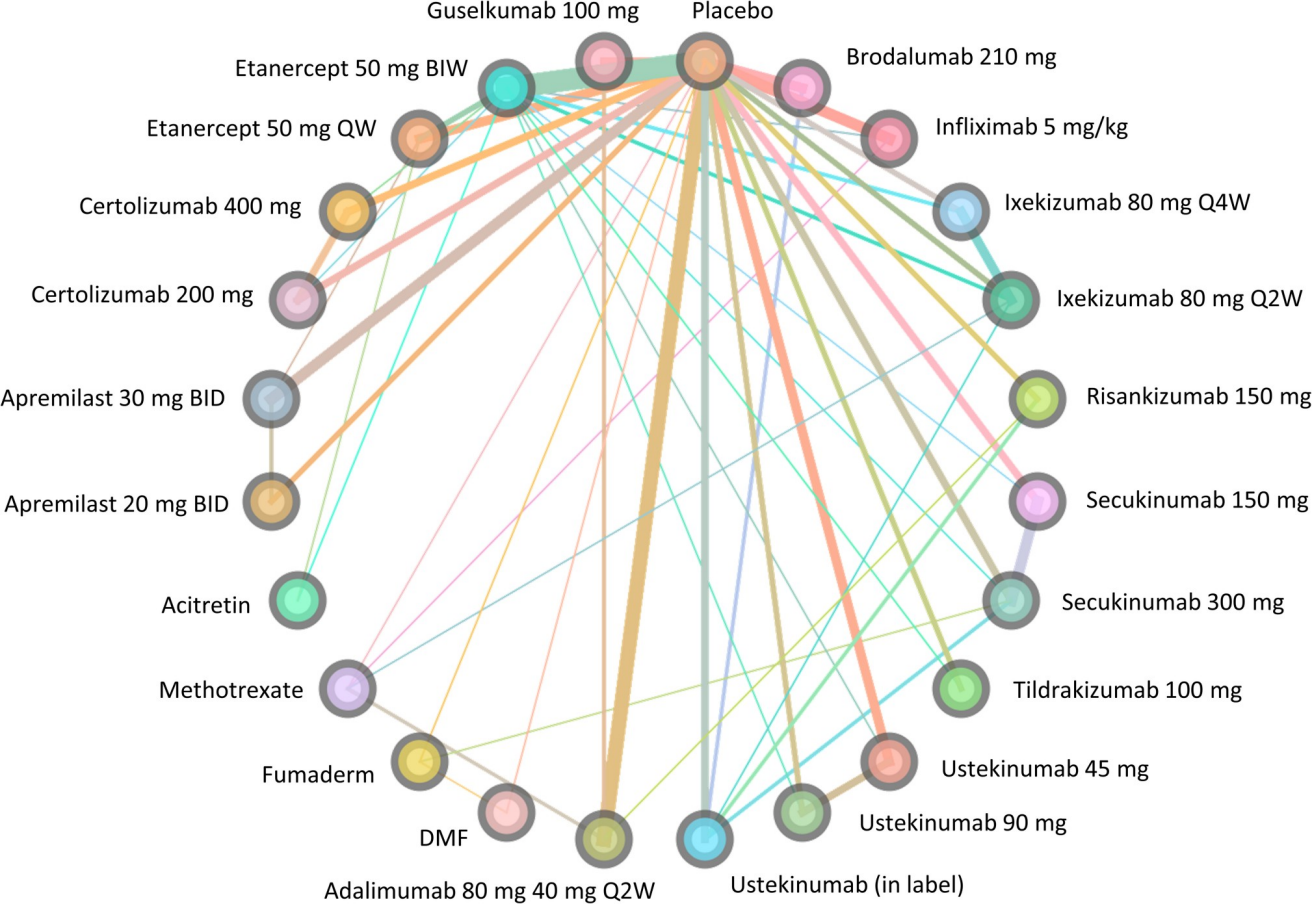
Network diagram for the base-case analysis.

Fig 2 displays the evidence network for the analysis of the included trials. Data were available on all interventions of interest.

### Study and patient characteristics

The main details of trials included in the NMA are summarised in Table 1. Patient eligibility criteria in the included studies were largely consistent, with most requiring patients to have baseline BSA ≥ 10% and PASI score of ≥ 10 or ≥ 12. However, three trials [60–63] only required a minimum BSA of 5%.

**Table 1:** Characteristics of trials included in the NMA.

Study	Study design			Interventions and comparator(s) (n)	Baseline characteristics				
	PASI outcomes reported	Primary endpoint (weeks)	Main inclusion criteria		Disease duration in years: mean (SD/ range*)	PsA (%)	Age in years: mean (SD/ range*)	Male (%)	% prior systemic therapy: conventional/ biologic
AMAGINE-1 [64]	50, 75, 90, 100	12	PASI ≥12, BSA ≥10%	Placebo (n = 220)	21 (12)	29	47 (13)	73	75/46
				Brodalumab 140 mg (n = 219)	19 (13)	27	46 (13)	74	65/45
				Brodalumab 210mg (n = 222)	20 (13)	26	46 (12)	73	70/47
AMAGINE-2 [65]	50, 75, 90, 100	12	PASI ≥12, BSA ≥10%	Placebo (n = 309)	18 (12)	17	44 (13)	71	74/29
				Brodalumab 140 mg (n = 610)	19 (12)	21	45 (13)	68	77/29
				Brodalumab 210 mg (n = 612)	19 (12)	19	45 (13)	69	77/29
				Ustekinumab in-label (n = 300)	19 (13)	17	45 (13)	68	75/28
AMAGINE-3 [65]	50, 75, 90, 100	12	PASI ≥12, BSA ≥10%	Placebo (n = 315)	18 (12)	19	44 (13)	66	65/24
				Brodalumab 140 mg (n = 629)	17 (12)	21	45 (13)	70	70/25
				Brodalumab 210 mg (n = 624)	18 (12)	20	45 (13)	69	68/25
				Ustekinumab in-label (n = 313)	18 (12)	20	45 (13)	68	70/24
Nakagawa 2016 [66]	75, 90, 100	12	PASI ≥12, BSA ≥10%	Placebo (n = 38)	17 (11)	18	47 (11)	71	NR/8
				Brodalumab 140 mg (n = 37)	15 (10)	16	46 (13)	81	NR/8
				Brodalumab 210 mg (n = 37)	15 (11)	14	46 (12)	78	NR/14
Papp 2012 [22]	50, 75, 90, 100	12	PASI ≥12, BSA ≥10%	Placebo (n = 38)	18 (12)	18	42 (14)	58	24/35
				Brodalumab 140 mg (n = 39)	19 (10)	28	44 (12)	72	41/35
				Brodalumab 210 mg (n = 40)	17 (10)	30	42 (12)	62	40/35
UNCOVER-1 [67]	50, 75, 90, 100	12	PASI ≥12, BSA ≥10%	Placebo (n = 431)	NR (NR)	NR	46 (13)	70	52/42
				Ixekizumab 80mg Q4W (n = 432)	NR (NR)	NR	46 (13)	67	49/39
				Ixekizumab 80mg Q2W (n = 433)	NR (NR)	NR	45 (12)	67	57/40
UNCOVER-2 [68]	75, 90, 100	12	PASI ≥10, BSA ≥10%	Placebo (n = 168)	19 (13)	NR	45 (12)	71	48/26
				Etanercept 50mg BIW (n = 358)	19 (12)	NR	45 (13)	66	48/21
				Ixekizumab 80mg Q4W (n = 347)	19 (13)	NR	45 (14)	70	51/25
				Ixekizumab 80mg Q2W (n = 351)	18 (12)	NR	45 (13)	63	51/24
UNCOVER-3 [68]	75, 90, 100	12	PASI ≥10, BSA ≥10%	Placebo (n = 193)	18 (13)	NR	46 (12)	71	43/17
				Etanercept 50mg BIW (n = 382)	18 (12)	NR	46 (14)	70	48/16
				Ixekizumab 80mg Q4W (n = 386)	18 (12)	NR	46 (13)	67	47/15
				Ixekizumab 80mg Q2W (n = 385)	18 (12)	NR	46 (13)	66	44/15
IXORA-S [69, 70]	75, 90, 100	12	PASI ≥10, BSA ≥NR	Ixekizumab 80 mg Q2W (n = 136)	18 (11)	NR	43 (13)	66	93/13
				Ustekinumab (45 mg if <100 kg; 90 mg if >100 kg) (n = 166)	18 (12)	NR	44 (13)	68	92/15
Reich 2017 [71]	75, 90, 100	12	PASI ≥NR BSA ≥NR	Ixekizumab 80 mg Q2W (n = 54)	NR (NR)	NR	NR (NR)	NR	0/0
				Methotrexate (n = 54)	NR (NR)	NR	NR (NR)	NR	0/0
IXORA-P [72]	75, 90, 100	12	PASI ≥12, BSA ≥10%	Ixekizumab 80mg Q4W (n = 310)	19 (12)	19	47 (14)	64	47/47
				Ixekizumab 80mg Q2W (n = 611)	20 (13)	16	49 (14)	67	48/49
Khattri 2018 [73]	50, 75, 90, 100	12	PASI ≥12, BSA ≥10%	Ixekizumab 80mg Q4W (n = 6)	22 (23)	NR	55 (7)	50	NR/NR
				Ixekizumab 80mg Q2W (n = 6)	17 (13)	NR	42 (13)	83	NR/NR
FEATURE [74]	50, 75, 90, 100	12	PASI ≥12, BSA ≥10%	Placebo (n = 59)	20 (14)	NR	47 (14)	66	49/44
				Secukinumab 150mg (n = 59)	20 (13)	NR	46 (15)	68	66/48
				Secukinumab 300mg (n = 59)	18 (12)	NR	45 (13)	64	34/39
ERASURE [75]	50, 75, 90, 100	12	PASI ≥12, BSA ≥10%	Placebo (n = 248)	17 (12)	27	45 (13)	69	44/29
				Secukinumab 150mg (n = 245)	18 (12)	19	45 (13)	69	51/30
				Secukinumab 300mg (n = 245)	17 (11)	23	45 (14)	69	52/29
FIXTURE [75]	50, 75, 90, 100	12	PASI ≥12, BSA ≥10%	Placebo (n = 326)	17 (12)	15	44 (13)	73	61/11
				Etanercept 50mg BIW (n = 326)	16 (12)	14	44 (13)	71	63/14
				Secukinumab 150mg (n = 327)	17 (12)	15	45 (13)	72	61/14
				Secukinumab 300mg (n = 327)	16 (12)	15	45 (13)	69	60/12
JUNCTURE [76]	50, 75, 90, 100	12	PASI ≥12, BSA ≥10%	Placebo (n = 61)	20 (12)	20	44 (13)	62	48/21
				Secukinumab 150mg (n = 61)	21 (15)	26	44 (14)	67	51/25
				Secukinumab 300mg (n = 60)	21 (14)	23	47 (14)	77	50/25
SCULPTURE [77]	50, 75, 90, 100	12	PASI ≥12, BSA ≥10%	Secukinumab 150mg (n = 482)	17 (13)	22	45 (13)	63	NR/27
				Secukinumab 300mg (n = 484)	17 (13)	19	47 (13)	69	NR/29
CLEAR [78]	75, 90, 100	12	PASI ≥12, BSA ≥10%	Secukinumab 300mg (n = 337)	20 (13)	21	45 (14)	68	65/14
				Ustekinumab (45 mg if <100 kg; 90 mg if >100 kg) (n = 339)	16 (11)	16	45 (14)	74	66/13
PRIME [79, 80]	50, 75, 90, 100	24	PASI ≥10, BSA ≥10%	Secukinumab 300mg (n = 105)	16 (13)	4	43 (14)	62	0/0
				Fumaderm (n = 97)	17 (13)	8	47 (13)	62	0/0
SIGNATURE [81]	75, 90	16	PASI ≥10, BSA NR	Secukinumab 150mg (n = 115)	NR (NR)	41	45 (NR)	55	NR/100
				Secukinumab 300mg (n = 118)	NR (NR)	40	47 (NR)	57	NR/100
CLARITY [82]	75, 90, 100	16	PASI ≥12, BSA ≥10%	Secukinumab (n = 550)	17 (12)	NR	45 (14)	65	NR/20
				Ustekinumab (n = 550)	17 (13)	NR	45 (14)	68	NR/24
VOYAGE 1 [30]	75, 90, 100	16	PASI ≥12, BSA ≥10%	Placebo (n = 174)	18 (12)	17	45 (13)	68	53/20
				Adalimumab 80 mg 40 mg Q2W (n = 334)	17 (11)	19	43 (13)	75	64/21
				Guselkumab 100 mg (n = 329)	18 (12)	20	44 (13)	73	64/22
VOYAGE 2 [31]	75, 90, 100	16	PASI ≥12, BSA ≥10%	Placebo (n = 248)	18 (12)	19	43 (12)	70	60/22
				Adalimumab 80 mg 40 mg Q2W (n = 248)	18 (12)	18	43 (12)	69	64/20
				Guselkumab 100 mg (n = 496)	18 (12)	18	44 (12)	70	67/20
Ohtsuki 2018 [83]	50, 75, 90, 100	16	PASI ≥12, BSA ≥10%	Placebo (n = 64)	14 (10)	16	48 (11)	84	59/16
				Guselkumab 100 mg (n = 63)	14 (9)	16	48 (11)	75	59/18
ORION [84]	90, 100	16	PASI ≥12, BSA ≥10%	Placebo (n = 16)	NR (NR)	NR	NR (NR)	NR	NR/NR
				Guselkumab 100 mg (n = 62)	NR (NR)	NR	NR (NR)	NR	NR/NR
IMMhance [85, 86]	90	16	PASI NR, BSA NR	Placebo (n = 100)	NR (NR)	NR	NR (NR)	NR	NR/NR
				Risankizumab 150mg (n = 407)	NR (NR)	NR	NR (NR)	NR	NR/NR
IMMvent [32, 33]	90, 100	16	PASI ≥12, BSA ≥10%	Adalimumab 80mg 40mg Q2W (n = 304)	NR (NR)	NR	NR (NR)	NR	NR/NR
				Risankizumab 150mg (n = 301)	NR (NR)	NR	NR (NR)	NR	NR/NR
UltiMMa-1 [35]	75, 90, 100	16	PASI ≥12, BSA ≥10%	Placebo (n = 102)	NR (NR)	35	49 (14)	77	NR/39
				Risankizumab 150mg (n = 304)	NR (NR)	28	48 (13)	70	NR/34
				Ustekinumab (in-label dose) (n = 100)	NR (NR)	23	47 (13)	70	NR/30
UltiMMa-2 [35, 36]	75, 90, 100	16	PASI ≥12, BSA ≥10%	Placebo (n = 98)	NR (NR)	33	46 (13)	68	NR/43
				Risankizumab 150mg (n = 294)	NR (NR)	25	46 (14)	69	NR/40
				Ustekinumab (in-label dose) (n = 99)	NR (NR)	27	49 (15)	67	NR/43
Papp 2015 [87]	75, 90	16	PASI ≥12, BSA ≥10%	Placebo (n = 46)	NR (NR)	24	46 (12)	83	NR/28
				Tildrakizumab 100mg (n = 89)	NR (NR)	17	46 (13)	85	NR/26
				Tildrakizumab 200mg (n = 86)	NR (NR)	17	43 (13)	76	NR/26
reSURFACE 1 [34]	75, 90, 100	12	PASI ≥12, BSA ≥10%	Placebo (n = 155)	NR (NR)	NR	48 (14)	65	NR/23
				Tildrakizumab 100mg (n = 309)	NR (NR)	NR	46 (13)	67	NR/23
				Tildrakizumab 200 mg (n = 308)	NR (NR)	NR	47 (13)	73	NR/23
reSURFACE2 [34]	75, 90, 100	12	PASI ≥12, BSA ≥10%	Placebo (n = 156)	NR (NR)	NR	46 (12)	72	NR/13
				Etanercept 50 mg BIW (n = 313)	NR (NR)	NR	46 (14)	71	NR/12
				Tildrakizumab 100 mg (n = 307)	NR (NR)	NR	45 (14)	72	NR/13
				Tildrakizumab 200 mg (n = 314)	NR (NR)	NR	45 (14)	72	NR/12
PEARL [88]	50, 75, 90, 100	12	PASI ≥12, BSA ≥10%	Placebo (n = 60)	14 (7)	12	40 (10)	88	72/15
				Ustekinumab 45mg (n = 61)	12 (8)	16	41 (13)	82	71/21
PHOENIX-1 [89]	50, 75, 90, 100	12	PASI ≥12, BSA ≥10%	Placebo (n = 255)	20 (12)	35	45 (11)	72	56/50
				Ustekinumab 45mg (n = 255)	20 (12)	29	45 (13)	69	55/53
				Ustekinumab 90mg (n = 256)	20 (11)	37	46 (11)	68	55/51
PHOENIX-2 [90]	50, 75, 90, 100	12	PASI ≥12, BSA ≥10%	Placebo (n = 410)	21 (12)	26	47 (13)	69	59/39
				Ustekinumab 45mg (n = 409)	19 (12)	26	45 (12)	69	55/38
				Ustekinumab 90mg (n = 411)	20 (12)	23	47 (12)	67	55/37
LOTUS [91]	50, 75, 90, 100	12	PASI ≥12, BSA ≥10%	Placebo (n = 162)	14 (9)	9	39 (12)	76	43/7
				Ustekinumab 45mg (n = 160)	15 (9)	9	40 (12)	78	39/12
Igarashi 2012 [92]	50, 75, 90	12	PASI ≥12, BSA ≥10%	Placebo (n = 32)	16 (11)	3	49 (NR)	84	66/0
				Ustekinumab 45mg (n = 64)	16 (8)	9	45 (NR)	83	73/2
				Ustekinumab 90mg (n = 62)	17 (11)	11	44 (NR)	76	84/0
CHAMPION [93]	50, 75, 90, 100	16	PASI ≥10, BSA ≥10%	Placebo (n = 53)	19 (9)	21	41 (11)	66	NR/NR
				Adalimumab 80 mg 40 mg Q2W (n = 108)	18 (10)	21	43 (13)	65	NR/NR
				Methotrexate (n = 110)	19 (10)	17	42 (12)	66	NR/NR
Goldminz 2015 [94]	75	16	PASI NC, BSA NC	Adalimumab 80 mg 40 mg Q2W (n = 15)	17 (NR)	13	51 (NR)	73	40/NR
				Methotrexate (n = 15)	22 (NR)	20	50 (NR)	87	27/NR
Cai 2016 [95]	75, 90, 100	12	PASI ≥NR, BSA ≥NR	Placebo (n = 87)	16 (10)	12	44 (13)	67	16/0
				Adalimumab 80 mg 40 mg Q2W (n = 338)	15 (10)	13	43 (12)	75	29/0
REVEAL [96]	75, 90, 100	16	PASI ≥12, BSA ≥12%	Placebo (n = 398)	18 (12)	28	45 (13)	65	22/13
				Adalimumab 80 mg 40 mg Q2W (n = 814)	18 (12)	28	44 (13)	67	23/12
Asahina 2010 [97]	50, 75, 90	16	PASI ≥12, BSA ≥12%	Placebo (n = 46)	16 (8)	NR	44 (11)	89	37/0
				Adalimumab 80 mg 40 mg Q2W (n = 43)	14 (7)	NR	44 (14)	81	42/0
Gordon 2006 [61]	75, 100	12	PASI ≥NR, BSA ≥5%	Placebo (n = 52)	19 (1–40)	31	43 (20–70)	65	NR/0
				Adalimumab 80 mg 40 mg Q2W (n = 46)	21 (1–58)	33	46 (20–71)	71	NR/0
X-PLORE [98]	75, 90, 100	16	PASI ≥12, BSA ≥10%	Placebo (n = 42)	18 (13)	29	47 (NR)	67	50/36
				Adalimumab 80 mg 40 mg Q2W (n = 43)	19 (13)	26	50 (NR)	70	40/60
CIMPACT [99]	75, 90	12	PASI ≥12, BSA ≥10%	Placebo (n = 57)	19 (13)	21	47 (13)	60	NR/19
				Certolizumab 200mg (n = 165)	20 (13)	16	47 (14)	69	NR/27
				Certolizumab 400mg (n = 167)	18 (12)	14	45 (12)	64	NR/29
				Etanercept 50mg BIW (n = 170)	17 (12)	16	45 (14)	75	NR/30
CIMPASI-1 [100]	75, 90, 100	16	PASI ≥12, BSA ≥10%	Placebo (n = 51)	19 (13)	8	48 (13)	69	NR/29
				Certolizumab 200mg (n = 95)	17 (12)	11	45 (13)	71	NR/32
				Certolizumab 400mg (n = 88)	18 (13)	17	44 (12)	68	NR/33
CIMPASI-2 [100]	75, 90, 100	16	PASI ≥12, BSA ≥10%	Placebo (n = 49)	15 (12)	18	43 (15)	53	NR/29
				Certolizumab 200mg (n = 91)	19 (14)	24	47 (13)	64	NR/35
				Certolizumab 400mg (n = 87)	19 (12)	30	46 (14)	49	NR/35
Reich 2012 [101]	50, 75, 90	12	PASI ≥12, BSA ≥10%	Placebo (n = 59)	20 (12)	NR	43 (13)	63	NR/24
				Certolizumab 200mg (n = 59)	21 (11)	NR	43 (10)	75	NR/22
				Certolizumab 400mg (n = 58)	20 (10)	NR	44 (12)	72	NR/24
Leonardi 2003 [102]	50, 75, 90	16	PASI ≥10, BSA ≥10%	Placebo (n = 166)	18 (12)	NR	46 (13)	63	NR/NR
				Etanercept 25 mg BIW (n = 162)	19 (12)	NR	45 (13)	67	NR/NR
				Etanercept 50 mg BIW (n = 164)	19 (12)	NR	45 (10)	65	NR/NR
Gottlieb 2003 [103]	50, 75, 90	12	PASI ≥NR, BSA ≥10%	Placebo (n = 55)	20 (2)	65	47 (18–77)	67	36/NR
				Etanercept 25 mg BIW (n = 57)	23 (2)	72	48.2 (25–72)	58	39/NR
Papp 2005 [104]	50, 75, 90	12	PASI ≥10, BSA ≥10%	Placebo (n = 193)	18 (1–52)	26	45 (11)	64	39/0
				Etanercept 25mg BIW (n = 196)	22 (1–65)	28	45 (12)	65	35/0
				Etanercept 50mg BIW (n = 194)	18 (1–61)	26	45 (12)	67	38/0
Van de Kerkhof 2008 [105]	50, 75, 90	12	PASI ≥10, BSA ≥10%	Placebo (n = 46)	17 (8)	11	44 (13)	54	48/0
				Etanercept 50mg BIW (n = 96)	19 (11)	16	46 (13)	62	49/0
Bagel 2012 [106]	50, 75, 90	12	PASI ≥10, BSA ≥10%	Placebo (n = 62)	11 (1–49)	NR	42 (8–70)	58	NR/7
				Etanercept 50mg BIW (n = 62)	18 (1–45)	NR	39 (18–71)	53	NR/7
Bachelez 2015 [107]	50, 75, 90	12	PASI ≥12, BSA ≥10%	Placebo (n = 107)	17 (1–57)	24	46 (21–81)	66	NR/11
				Etanercept 50mg BIW (n = 335)	18 (1–62)	21	42 (18–74)	70	NR/11
Tyring 2006 [108]	50, 75, 90	12	PASI ≥10, BSA ≥10%	Placebo (n = 309)	20 (11)	33	46 (12)	70	NR/NR
				Etanercept 50mg BIW (n = 311)	20 (12)	35	46 (13)	65	NR/NR
PRISTINE [109]	50, 75, 90	12	PASI ≥10, BSA ≥10%	Etanercept 50mg QW (n = 137)	17 (11)	29	44 (13)	74	NR/NR
				Etanercept 50mg BIW (n = 136)	18 (10)	33	44 (13)	65	NR/NR
M10-114 [110]	75, 90, 100	12	PASI ≥12, BSA ≥10%	Placebo (n = 68)	19 (13)	21	44 (14)	69	28/15
				Etanercept 50mg BIW (n = 141)	17 (13)	23	43 (13)	70	26/14
M10-315 [111]	75, 90, 100	12	PASI ≥12, BSA ≥10%	Placebo (n = 72)	16 (12)	21	45 (14)	64	28/4
				Etanercept 50mg BIW (n = 139)	15 (12)	33	45 (15)	61	32/8
PIECE [112]	50, 75, 90, 100	12	PASI ≥10, BSA ≥10%	Etanercept 50mg BIW (n = 23)	18 (11)	13	4 (13)	57	100/22
				Infliximab 5mg/kg (n = 25)	22 (13)	8	46 (14)	72	96/12
ACCEPT [113]	75, 90	12	PASI ≥12, BSA ≥10%	Etanercept 50mg BIW 45mg (n = 347)	19 (12)	27	46 (13)	71	57/12
				Ustekinumab 45mg (n = 209)	19 (12)	30	45 (13)	64	62/12
				Ustekinumab 90mg (n = 347)	19 (12)	27	45 (12)	67	52/10
Caproni 2009 [114]	50, 75	12	PASI ≥10, BSA ≥10%	Etanercept 50mg BIW (n = 30)	NR (NR)	NR	NR (28–67)	43	NR/NR
				Acitretin (n = 30)	NR (NR)	NR	NR (31–65)	37	NR/NR
Gisondi 2008 [115]	50, 75	12	PASI ≥NR, BSA ≥NR	Etanercept 25mg BIW (n = 22)	24 (11)	NR	55 (11)	55	NR/0
				Acitretin (n = 20)	19 (16)	NR	55 (11)	60	NR/0
Yang 2012 [116]	75, 90, 100	10	PASI ≥12, BSA ≥10%	Placebo (n = 45)	16 (9)	NR	40 (11)	78	NR/NR
				Infliximab 5mg/kg (n = 84)	16 (11)	NR	39 (12)	71	NR/NR
EXPRESS [117]	50, 75, 90, 100	10	PASI ≥12, BSA ≥10%	Placebo (n = 77)	17 (11)	29	44 (13)	79	46/0
				Infliximab 5mg/kg (n = 301)	19 (11)	31	43 (12)	69	42/0
Chaudhari 2001 [118]	75	10	PASI ≥NR, BSA ≥5%	Placebo (n = 11)	NR (NR)	NR	45 (12)	NR	NR/0
				Infliximab 5mg/kg (n = 11)	NR (NR)	NR	51 (14)	NR	NR/0
SPIRIT [119]	50, 75, 90	10	PASI ≥12, BSA ≥10%	Placebo (n = 51)	16 (IQR: 6, 22)	33	45 (IQR: 30, 52)	61	82/31
				Infliximab 5mg/kg (n = 99)	16 (IQR: 10, 25)	29	44 (IQR: 34, 53)	74	89/33
EXPRESS II [120]	75, 90	10	PASI ≥10, BSA ≥10%	Placebo (n = 208)	NR (NR)	26	44 (13)	69	NR/13
				Infliximab 5mg/kg (n = 314)	NR (NR)	28	45 (13)	65	NR/14
Torii 2010 [121]	50, 75, 90	10	PASI ≥12, BSA ≥10%	Placebo (n = 19)	11 (6)	37	43 (12)	74	95/NR
				Infliximab 5mg/kg (n = 35)	14 (9)	29	47 (13)	63	94/NR
RESTORE1 [122]	50, 75, 90	16	PASI ≥12, BSA ≥10%	Infliximab 5mg/kg (n = 653)	19 (12)	NR	44 (18–78)	67	61/8
				Methotrexate (n = 215)	17 (10)	NR	42 (18–69)	69	65/8
PSOR-005 [123]	50, 75, 90, 100	16	PASI ≥12, BSA ≥10%	Placebo (n = 88)	20 (12)	19	44 (14)	60	44/NR
				Apremilast 20 mg BID (n = 87)	19 (12)	18	45 (13)	63	49/NR
				Apremilast 30 mg BID (n = 88)	19 (12)	24	44 (15)	57	53/NR
ESTEEM 1 [124]	50, 75, 90	16	PASI ≥12, BSA ≥10%	Placebo (n = 282)	19 (12)	NR	47 (13)	69	36/28
				Apremilast 30 mg BID (n = 562)	20 (13)	NR	46 (13)	67	38/29
ESTEEM 2 [125]	50, 75, 90	16	PASI ≥12, BSA ≥10%	Placebo (n = 137)	19 (12)	NR	46 (13)	73	39/32
				Apremilast 30 mg BID (n = 274)	18 (11)	NR	45 (13)	64	39/34
Papp 2013 [126]	50, 75, 90	12	BSA ≥10%	Placebo (n = 87)	NR (NR)	13	44 (12)	61	NR/NR
				Apremilast 20 mg BID (n = 85)	NR (NR)	25	48 (12)	58	NR/NR
Ohtsuki 2017 [127]	50, 75, 90	16	PASI ≥12, BSA ≥10%	Placebo (n = 84)	12 (9)	NR	48 (12)	74	26/5
				Apremilast 20 mg BID (n = 85)	13 (11)	NR	52 (13)	81	40/4
				Apremilast 30 mg BID (n = 85)	14 (9)	NR	52 (13)	84	31/2
LIBERATE [128, 129]	50, 75, 90	16	PASI ≥12, BSA ≥10%	Placebo (n = 84)	17 (12)	NR	43 (15)	70	83/0
				Apremilast 30 mg BID (n = 83)	20 (13)	NR	46 (14)	59	80/0
				Etanercept 50 mg QW (n = 83)	18 (12)	NR	47 (14)	59	70/0
UNVEIL [62, 63, 130]	75	16	PASI ≥NR, BSA ≥5%	Placebo (n = 73)	14 (13)	NR	51 (14)	56	0/0
				Apremilast 30mg BID (n = 148)	18 (14)	NR	49 (15)	50	0/0
BRIDGE [131]	50, 75, 90	16	PASI ≥10, BSA ≥10%	Placebo (n = 138)	NR (NR)	NR	44 (14)	68	MTX: 10 CIC: 6; ACI: 7/TNF: 2
				Dimethyl Fumarate (n = 208)	NR (NR)	NR	44 (15)	62	MTX: 7; CIC: 4; ACI: 3/TNF: 2.5; IL: 1
				Fumaderm (n = 286)	NR (NR)	NR	45 (14)	65	MTX: 14; CIC: 3; ACI: 5/TNF: 2; IL: 1

n, number of patients; PASI, Psoriasis Area and Severity Index; BSA, body surface area; PsA, Psoriatic Arthritis; SD, standard deviation; BID, twice daily; BIW, twice weekly; QW, weekly; Q2W, every 2 weeks; Q4W, every 4 weeks; mg, milligrams; kg, kilograms; NR, not reported; NC, not considered; MTX, methotrexate; CIC, cyclosporin; ACI, acitretin; TNF, tumour necrosis factor; IL, interleukin

* range reported only if SD not available

Patient characteristics were broadly similar across studies. In the majority of RCTs mean baseline PASI scores ranged from 8 [62, 63, 115] to 32 [121]. In the included trials, patients’ mean age ranged from 40 [116] to 57 years [59] and the percentage of males was between 40% [114] and 85% [97]. Exposure to prior therapies varied across studies. Three RCTs included only systemic-naïve patients [62, 71, 79], while in the remaining studies the mean percentage of patients who had received prior conventional systemic therapy ranged from 23% [96] to 98% [112]. The proportion of patients who received prior phototherapy also varied considerably across trials and, where reported, percentages ranged between 16% [96] and 98% [132].

When studies reported previous exposure to biologic therapy, this ranged from 1% [92] to 100% [81] across trials. More recent studies [64, 65, 67, 74, 89, 90] included greater proportions of patients with previous exposure to biologic therapies, whereas, other studies required patients to be naïve to biologic therapies [61, 62, 71, 79, 93–95, 97, 104, 105, 115, 117, 118, 128].

### Risk of bias

Overall, the risk of bias for most of the studies was judged to be low (details reported in S5 Table). Studies generally reported appropriate randomisation and allocation concealment. Patient characteristics appeared similar between treatment arms at baseline. Most of the studies were double-blind, however in some cases only investigator blinding was undertaken. Losses to follow-up appeared to be balanced between study arms, except for PRIME [79], where proportionally more patients were lost to follow-up in the FAE arm compared to secukinumab; Ohtsuki 2018 [83], where more patients dropped out of the placebo arm compared to guselkumab arms; and a phase II certolizumab trial [101, 133], where drop-outs from the placebo arm were much higher than from certolizumab arms. The risk of reporting bias was considered low in all studies. Most studies used an intention-to-treat or modified intention-to-treat approach, including all randomised patients. There was, however, variation in the method used to handle missing data: forty-one (53%) studies used non-responder imputation, 18 (23%) used last observation carried forward, four (5%) used multiple imputation methods, and the remaining fourteen (18%) did not report the method used.

### Model fit

Goodness of fit was assessed for the model with placebo-adjustment compared to the model without adjustment. The estimated placebo arm adjustment coefficient was statistically significant (-0.676, 95% CrI: -0.824 to -0.507), indicating that the adjustment reduced unexplained heterogeneity and improved the model. In addition, the 95% CrI of the random effect, τ, was estimated to be from 60.4 to 271.9 in the adjusted model compared with 52.5 to 466.8 in the unadjusted model. The narrowing of the 95% CrI in the adjusted model demonstrates a reduction in the between-study heterogeneity being captured by the adjustment coefficient. No significant inconsistency was identified in the base-case or sensitivity analysis networks.

### Efficacy relative to placebo

**Fig 3:**
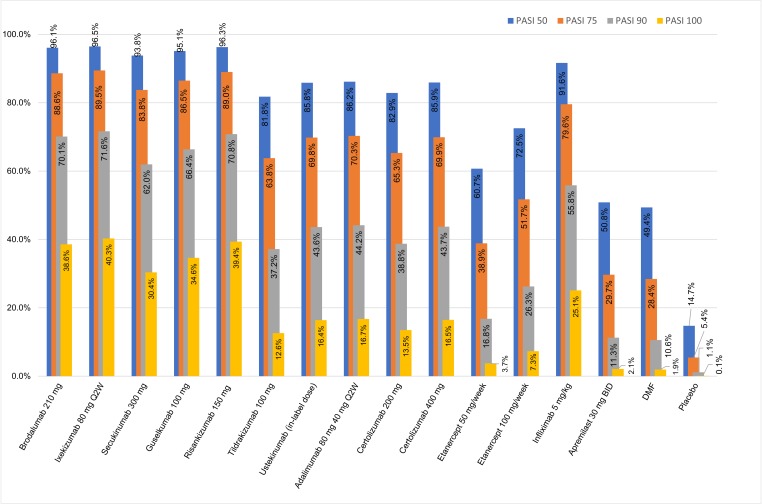
PASI response at end of induction—placebo-adjusted.

Fig 3 presents the probabilities of achieving each level of PASI response for each treatment. As shown in Table 2, the placebo-adjusted base-case analysis indicated all therapies to be superior to placebo. In comparison with placebo, licensed doses of ixekizumab, and brodalumab along with risankizumab and guselkumab have shown the greatest treatment effects at inducing all levels of PASI response. The effects of these treatments were particularly pronounced for PASI 100, for which the proportion achieving complete clearance was more than 350 times higher than with placebo. Compared with placebo, the number needed to treat (NNT) to achieve complete clearance was the lowest for ixekizumab (NNT = 3), risankizumab (NNT = 3), brodalumab (NNT = 3) and guselkumab (NNT = 3). Etanercept, apremilast and DMF were associated with the lowest probabilities of response and highest NNTs of 28, 50 and 55, respectively.

**Table 2:** Treatment effects at each level of PASI response for all interventions versus placebo at the end of induction. Number needed to treat included for PASI 100 –placebo-adjusted base-case.

Class	Treatment	Risk ratio versus placebo, median (95% CrI)				NNT for PASI 100 (95% CrI)
		PASI 50	PASI 75	PASI 90	PASI 100	
IL-17RA	Brodalumab 210mg	6.52 (3.39 to 14.63)	16.26 (7.07 to 42.54)	62.34 (22.61 to 188.04)	398.09 (124.07 to 1348.07)	2.60 (1.71, 4.72)
IL-17A	Ixekizumab 80mg Q2W	6.54 (3.39 to 14.75)	16.42 (7.10 to 43.31)	63.73 (22.91 to 194.67)	415.97 (127.94 to 1436.99)	2.49 (1.66, 4.43)
	Secukinumab 300mg	6.37 (3.36 to 13.91)	15.36 (6.89 to 38.40)	55.07 (21.08 to 155.69)	313.68 (104.99 to 976.56)	3.30 (2.02, 6.53)
IL-23	Guselkumab 100 mg	6.45 (3.37 to 14.30)	15.85 (6.99 to 40.62)	58.89 (21.90 to 173.16)	356.00 (114.80 to 1173.71)	2.90 (1.83, 5.52)
	Risankizumab 150 mg	6.53 (3.39 to 14.68)	16.32 (7.08 to 42.86)	62.89 (22.72 to 191.53)	405.52 (125.87 to 1392.95)	2.55 (1.69, 4.62)
	Tildrakizumab 100 mg	5.55 (3.15 to 10.77)	11.67 (5.90 to 24.89)	32.96 (15.02 to 76.80)	129.52 (54.00 to 327.55)	8.00 (3.81, 20.84)
IL-12/23	Ustekinumab (in-label dose)	5.82 (3.23 to 11.73)	12.80 (6.25 to 28.47)	38.74 (16.84 to 94.52)	168.83 (66.89 to 444.05)	6.14 (3.17, 14.57)
Anti-TNF	Adalimumab 40mg Q2W	5.84 (3.23 to 11.83)	12.88 (6.27 to 28.87)	39.22 (16.98 to 96.43)	172.50 (67.93 to 456.20)	6.01 (3.13, 14.09)
	Certolizumab 200mg	5.61 (3.17 to 11.02)	11.96 (6.00 to 25.72)	34.34 (15.43 to 80.91)	138.72 (56.63 to 355.49)	7.47 (3.61, 19.40)
	Certolizumab 400mg	5.82 (3.23 to 11.74)	12.80 (6.24 to 28.54)	38.74 (16.76 to 95.33)	169.03 (66.05 to 455.17)	6.11 (3.11, 14.93)
	Etanercept 50 mg / week	4.11 (2.66 to 6.76)	7.10 (4.30 to 12.37)	14.86 (8.45 to 27.43)	38.52 (20.58 to 75.53)	27.40 (10.16, 92.68)
	Etanercept 100 mg / week	4.92 (2.96 to 8.83)	9.50 (5.22 to 18.28)	23.35 (11.87 to 47.69)	75.41 (36.11 to 161.84)	13.87 (6.00, 39.80)
	Infliximab 5mg/kg	6.22 (3.32 to 13.23)	14.58 (6.71 to 35.10)	49.60 (19.72 to 133.53)	258.53 (91.49 to 765.11)	4.00 (2.31, 8.48)
PDE4	Apremilast 30mg BID	3.44 (2.38 to 5.25)	5.42 (3.54 to 8.70)	9.96 (6.16 to 16.86)	21.66 (12.65 to 39.09)	49.66 (16.63, 187.54)
FAE	Dimethyl Fumarate	3.32 (2.26 to 5.31)	5.14 (3.22 to 8.96)	9.27 (5.17 to 18.03)	19.69 (9.46 to 43.84)	54.68 (16.36, 238.67)

A median risk ratio higher than 1 indicates that active treatments are more efficacious than placebo at inducing a given level of PASI response. For NNT the exact results of the NMA are provided here for accuracy, although for interpretation the results are rounded up to provide information on the number of patients who would need to be treated in order to observe an additional PASI 100 response.

BID, twice daily; CrI, credible interval; DMF, dimethyl fumarate; PASI, Psoriasis Area and Severity Index; Q2W, every 2 weeks; Q4W, every 4

Weeks; NNT, number needed to treat.

### IL-17s versus IL-23s and IL-12/23

As shown in Fig 3, treatment with the IL-17RA brodalumab, the IL-17A ixekizumab and the IL-23s risankizumab and guselkumab produced similar proportions of patients achieving each level of PASI response, with any differences failing to reach statistical significance (PASI 75, 90 and 100 shown in Table 3). In the analysis risankizumab was significantly more efficacious than secukinumab, as were brodalumab and ixekizumab. Median point estimates suggest that guselkumab may be slightly more efficacious than secukinumab, but differences were not statistically significant. The NMA also showed all IL-17 targeted therapies, as well as guselkumab and risankizumab to be more efficacious than tildrakizumab, ustekinumab, adalimumab and certolizumab, which were all more efficacious than etanercept, apremilast and DMF. Risk ratios for each pairwise comparison at each level of PASI response are presented in S6 Table.

**Table 3:** Risk ratios for each pairwise comparison of IL-17As or IL-17RA vs IL-23 or IL-12/23 therapies at PASI 75, 90 and 100 from placebo-adjusted base-case analysis: median risk ratio (95% credible interval).

Intervention vs.	Comparator	Median risk ratio (95% credible interval)		
		PASI 75	PASI 90	PASI 100
Brodalumab	Ixekizumab	0.99 (0.95, 1.03)	0.98 (0.9, 1.06)	0.96 (0.81, 1.12)
	Secukinumab	**1.06 (1.02, 1.14)**	**1.13 (1.04, 1.28)**	**1.26 (1.08, 1.55)**
	Guselkumab	1.02 (0.98, 1.09)	1.05 (0.96, 1.19)	1.11 (0.93, 1.37)
	Risankizumab	1 (0.95, 1.04)	0.99 (0.9, 1.09)	0.98 (0.82, 1.17)
	Tildrakizumab	**1.39 (1.18, 1.78)**	**1.88 (1.45, 2.67)**	**3.03 (2.08, 4.86)**
	Ustekinumab (in label)	**1.27 (1.13, 1.52)**	**1.6 (1.32, 2.07)**	**2.35 (1.78, 3.29)**
Ixekizumab	Secukinumab	**1.07 (1.02, 1.15)**	**1.15 (1.06, 1.32)**	**1.32 (1.13, 1.63)**
	Guselkumab	1.03 (0.99, 1.1)	1.08 (0.99, 1.22)	1.16 (0.97, 1.44)
	Risankizumab	1.01 (0.97, 1.05)	1.01 (0.93, 1.11)	1.03 (0.86, 1.23)
	Tildrakizumab	**1.4 (1.18, 1.81)**	**1.92 (1.47, 2.75)**	**3.18 (2.16, 5.12)**
	Ustekinumab (in label)	**1.28 (1.13, 1.55)**	**1.64 (1.34, 2.15)**	**2.45 (1.83, 3.51)**
Secukinumab	Guselkumab	0.97 (0.91, 1.02)	0.94 (0.83, 1.04)	0.88 (0.72, 1.07)
	Risankizumab	**0.94 (0.87, 0.98)**	**0.88 (0.77, 0.96)**	**0.78 (0.62, 0.93)**
	Tildrakizumab	**1.31 (1.14, 1.62)**	**1.66 (1.33, 2.24)**	**2.39 (1.73, 3.6)**
	Ustekinumab (in label)	**1.2 (1.09, 1.37)**	**1.42 (1.22, 1.72)**	**1.85 (1.49, 2.41)**
Guselkumab	Risankizumab	0.97 (0.91, 1.01)	0.94 (0.83, 1.03)	0.88 (0.71, 1.05)
	Tildrakizumab	**1.35 (1.16, 1.71)**	**1.77 (1.39, 2.47)**	**2.72 (1.89, 4.27)**
	Ustekinumab (in label)	**1.24 (1.11, 1.46)**	**1.51 (1.27, 1.93)**	**2.1 (1.6, 2.94)**
Risankizumab	Tildrakizumab	**1.39 (1.18, 1.79)**	**1.89 (1.45, 2.71)**	**3.09 (2.1, 4.99**
	Ustekinumab (in label)	**1.27 (1.13, 1.53)**	**1.62 (1.33, 2.11)**	**2.39 (1.79, 3.42)**
Tildrakizumab	Ustekinumab (in label)	0.92 (0.8, 1.01)	0.86 (0.69, 1.02)	0.77 (0.56, 1.03)

A median risk ratio higher than 1 indicates that the intervention is more efficacious than the comparator at inducing a given level of PASI response.

In terms of NNT compared to ustekinumab, five patients would need to be treated with brodalumab, ixekizumab or risankizumab, six with guselkumab and eight with secukinumab for one more patient to achieve complete clearance. Compared to secukinumab, eleven, twelve and thirteen patients would need to be treated with ixekizumab, risankizumab and brodalumab, respectively, in order to generate an additional PASI 100 responder.

### Additional analyses

The analysis that did not adjust for cross-trial variation in placebo responses produced results similar to the placebo-adjusted analysis, though the model fit was poorer, indicating that the results are at risk of heterogeneity-induced bias. For completeness, details of this analysis can be found in S7 Table.

Thirty studies [32, 61, 62, 71, 73, 79, 84, 85, 92–95, 97, 102–105, 108, 109, 114–118, 121, 123, 126–128, 131], were excluded from the network in the sensitivity analysis which restricted trial inclusion to those that reported a minimum of 5% of patients with prior biologic exposure. Among these studies were all five studies [102–105, 109] evaluating the lower dose of etanercept (50 mg weekly) and the only study evaluating DMF [131]. For all other comparators, the results were similar to those of the placebo adjusted base-case. Details of this analysis can be found in S8 Table.

A sensitivity analysis was carried out to test the effect of using 12-week rather than 16-week outcomes from three secukinumab trials (CLEAR [78], SIGNATURE [81] and CLARITY [82]). To test for small study bias, a further sensitivity analysis was carried out excluding the eighteen trials with fewer than 50 patients per trial arm [73, 84, 85, 100, 134–147]. There were no major differences between the results of these sensitivity analyses and the base-case analysis.

## Discussion

Our NMA evaluating PASI response among licensed treatments for moderate-to-severe plaque psoriasis included 77 RCTs and a total of 34,816 patients. Data were available on all interventions set out in the inclusion criteria. The base-case analysis showed that all active treatments were superior to placebo. A comparison of PASI response between the classes of drugs with the highest efficacy, IL-17, IL-23 and IL-12/23 targeted treatments, was also undertaken. This showed that IL-17 inhibitors (brodalumab, ixekizumab and secukinumab) were more efficacious than tildrakizumab and ustekinumab and not significantly different from guselkumab. Compared to risankizumab, there was no significant difference in our analysis for brodalumab and ixekizumab, however secukinumab was less efficacious. Comparisons within the IL-17 inhibitor class showed that ixekizumab and brodalumab were not significantly different from one another, but that both were more efficacious than secukinumab. When comparisons were made among the IL-23 inhibitors, risankizumab and guselkumab were both more efficacious than tildrakizumab, but were not significantly different from one another. The greatest benefits of IL-17 inhibitors and IL-23 inhibitors guselkumab and risankizumab were seen for PASI 90 and PASI 100 response.

Since 2017, numerous NMAs have been published comparing the efficacy of various treatments in moderate to severe plaque type psoriasis [23, 24, 38, 148–153], and some have been critically appraised in the literature [154]. This largely reflects the dynamic development of the field, both in terms of treatment availability and new clinical trials being carried out. However, none of the currently published NMAs includes all currently licensed treatments in this patient population. In addition to NMAs, several pairwise meta-analyses have been undertaken comparing the efficacy of treatments in moderate-to-severe plaque psoriasis [155–157]. However, pairwise meta-analyses can only provide limited information and do not allow for a formal comparison of all available treatments. Valid relative efficacy comparisons between multiple treatments can only be made in an NMA [158, 159].

To our knowledge, this NMA provides the most recent and comprehensive comparison of available treatments for chronic plaque psoriasis. Specifically, it is the first to include the recently published clinical trial data for risankizumab and certolizumab pegol. Though many of the most recent clinical trials in moderate-to-severe psoriasis include a head-to-head comparison of targeted therapies, there are still many comparisons that have not been observed. In their absence, the network meta-analytic approach gives the most reliable estimate of comparative efficacy [159].

This NMA was based on a systematic review of RCTs evaluating licensed treatment doses. We followed a protocol designed for the systematic review; however, this was not registered online. English-language publications were searched, which has the potential to introduce bias [160, 161]; however, we believe this was unlikely to have had a substantial impact on our findings. A recent systematic review [23] that did not apply language restrictions identified no additional studies that would have been relevant to our NMA.

Although we were interested in evaluating licensed treatments, we included trials with any intervention or dose as the comparator. Although other recent NMAs chose to restrict inclusion to licensed doses only [25], we believe that such a broad approach allowed the construction of an extensive network of evidence and ensured that all relevant evidence was included and uncertainty in efficacy estimates was reduced [158].

As we set out to provide the best efficacy estimates to inform clinical practice, our analysis was dose-specific. Some recent NMAs have analysed a broad range of treatments, however the conclusions that can be drawn from these may be limited by their design. In particular, some of these analysis pool together both licensed and unlicensed doses of treatments [23, 24]. Although this has the advantage of including all the available information, interpretation of results may be challenging, and may lead to underestimation of the efficacy of treatments available to patients if evidence for subtherapeutic doses is included. Therefore, we analysed different doses separately to provide information we believe to be most relevant to clinical practice. Other recent NMAs [148, 149] have analysed a narrower range of treatments which makes a comprehensive assessment of the available treatments for plaque psoriasis impossible and conclusions limited in their relevance.

We evaluated all treatments at the end of their licensed induction period, which varies by drug [29, 162–172]. As a result, and similarly to a number of other NMAs [25, 38, 173], we combined data from different timepoints (10–16 weeks). Although this may have introduced some heterogeneity, it allowed the inclusion of all relevant information. In the recent Cochrane review and NMA [23], only trials with follow up between 12 and 16 weeks were included. Though this may have reduced the between-study heterogeneity, it resulted in the exclusion of all the pivotal studies comparing infliximab to placebo [116–121].

As our NMA only included data up to 16 weeks obtained from clinical trials, it does not provide an estimate of long-term effectiveness in a trial or real-world clinical setting. Psoriasis is a chronic condition, therefore a better understanding of the comparative effectiveness of biological drugs beyond induction would be beneficial. The randomised evidence is however limited, although some trials have reported 52-week results [30, 35, 36, 65, 69, 75, 174, 175] and researchers have synthesised some of these data [176]. Existence of such data will facilitate future evaluation of these treatments using an NMA approach.

A further limitation of the analysis was the inclusion of only PASI response rates. Although these are the most commonly used efficacy outcomes in psoriasis clinical trials, they do not provide a complete understanding of patient wellbeing. Statistical analysis of health-related quality of life (HRQoL) and safety data may also be helpful in clinical decision making. One of the most recent evaluations of HRQoL and tolerability in psoriasis suggested that IL-17 and IL-23 inhibitors provide the most benefit in terms of quality of life, while the occurrence of serious adverse events is not significantly more frequent than in patients treated with placebo [23, 24].

In psoriasis, placebo response rates have been observed to vary considerably between trials [44], reflecting differences in study design and patient characteristics. In line with these observations, we have identified variation in factors such as patient age, baseline PASI scores, or prior exposure to systemic therapy. To account for the potential heterogeneity introduced by these differences, our analysis presented the results of two statistical models: one that adjusted for variation in placebo-arm response and one that did not. Consistent with three previous NMAs [25, 38, 51], the estimated placebo arm adjustment coefficient was statistically significant, indicating that the adjustment reduced unexplained heterogeneity and improved the model. Cameron et al. explored several covariates in meta-regression analyses, including placebo-response adjustment, prior biologic use, body weight, disease duration, age, race and baseline outcome scores. In their analysis of PASI responses, the authors also found the placebo-adjusted model to have the best fit and argued that it likely accounted for multiple effect modifiers simultaneously.

Our results are broadly similar to those of other recently published NMAs [23–25, 38] in showing that all active treatments are superior to placebo and that IL-17 (in particular brodalumab and ixekizumab) and IL-23 inhibitors (in particular guselkumab and risankizumab) are the most efficacious in inducing PASI response. All analyses show that achieving complete and near complete clearance is more likely with these new treatments. We can also compare our results to those of the ECLIPSE study which has recently been reported in Johnson & Johnson press material [37]. Both our analysis and the current reports for ECLIPSE suggest there is no significant difference between secukinumab and guselkumab at the end of a 12-week induction period. Future work should focus on incorporating this new head-to-head comparison once it is published.

We believe our NMA adds to the current body of evidence, evaluating the efficacy of licensed doses of all treatments that are or will soon be available to patients. This NMA is the first to include risankizumab, and the recently licensed certolizumab pegol in its licensed dose. As our searches have been carried out recently, we have also included a number of recent trials that were not previously included in any NMA [72, 73, 79, 81–84].

With PASI 90 and PASI 100 becoming attainable treatment goals, consensus is growing that dermatologists should expect more highly effective therapies for their psoriasis patients [177, 178], especially as these have been shown to translate to improved HRQoL [179, 180].

## Conclusions

The results of the NMA indicated that brodalumab, ixekizumab, risankizumab and guselkumab showed the highest levels of efficacy. It is not possible to make conclusions at drug class level, as there are differences between treatments within the same class, some significant. In our analysis the IL-17RA inhibitor brodalumab and IL-17A inhibitor ixekizumab as well as the IL-23 targeted risankizumab were found to be significantly more efficacious than secukinumab. The NMA showed that across all levels of PASI response, all IL-17 targeted therapies, risankizumab and guselkumab outperformed tildrakizumab and ustekinumab as well as anti-TNFs such as adalimumab, certolizumab pegol and etanercept. These effects were particularly pronounced for PASI 90 and PASI 100. Our results are broadly consistent with previously published NMAs.

## Supporting information

S1 Text Protocol. (DOCX)

S2 Text Model. (DOCX)

S1 Table Search strategies. (XLSX)

S2 Table Study eligibility criteria. (DOCX)

S3 Table PASI outcomes as reported in trials included in the NMA. (XLSX)

S4 Table Excluded studies. (XLSX)

S5 Table Critical appraisal checklist for studies included in NMA. (DOCX)

S6 Table RRs for pairwise comparisons of PASI 50, 75, 90 and 100. (DOCX)

S7 Table Results of NMA without placebo adjustment. (DOCX)

S8 Table Results of sensitivity analysis. (DOCX)

S9 Table PRISMA checklist. (DOCX)
