# Supplementary material for: Assessing the relative efficacy of interleukin-17 and interleukin-23 targeted treatments for moderate-to-severe plaque psoriasis: A systematic review and network meta-analysis of PASI response
Source: PLoS One. 2019 Aug 14;14(8):e0220868. doi: 10.1371/journal.pone.0220868 (PMC6693782; doi:10.1371/journal.pone.0220868)
Supplement: S1 Text — (DOCX) [file pone.0220868.s001.docx]

Assessing the relative efficacy of interleukin-17 and interleukin-23 targeted treatments for moderate-to-severe plaque psoriasis: a systematic review and network meta-analysis of PASI response

*Laura Sawyer, Kinga Malottki, Celia Sabry-Grant, Najeeda Yasmeen, Emily Wright, Anne Sohrt, Emma Borg, Richard B Warren*

Review objective

The project objective is to systematically identify all RCT evidence regarding the efficacy and safety of biologic therapies for the treatment of moderate-to-severe plaque psoriasis. If possible, the RCTs will be combined in a network meta-analysis (NMA).

Searches

**Electronic databases**

The following electronic databases will be searched:

• Embase

• Medline, Medline In-Process and other non-indexed citations

• The Cochrane Library

**Supplementary searches**

To ensure the most recent published data are included, the following congresses will be searched:

• International Society for Pharmacoeconomics and Outcomes Research (ISPOR)

- US and EU congresses

• International Congress on Psoriasis

• World Congress of Dermatology (WCD)

• European Academy of Dermatology and Venereology (EADV)

• American Academy of Dermatology (AAD)

• British Association of Dermatologists Annual Meeting

Full search strategies have been developed and take into account variations in subject headings across databases. Briefly, the searches comprise of:

- Psoriasis disease terms
  - No disease severity restrictions have been placed on the search
- Therapies of interest
  - Compound and, where possible, marketed names
- Study design filters
  - RCTs
- Limited to:
  - Publication dated 2000 – current
  - Publications that are not case reports, letters or editorials
  - English language publications and human studies

Types of study to be included

Randomised controlled trials of any phase. Identified systematic reviews and NMAs will be ordered in full text and lists of included studies will be reviewed to identify any additional relevant publications.

All other types of studies (non-randomised studies, long-term extensions, editorials, case reports, reviews etc.) will be excluded.

Condition or domain being studied

Moderate-to-severe chronic plaque psoriasis

Participants/population

Adult patients with moderate-to-severe chronic plaque-type psoriasis will be included.

Paediatric patients, patients with non-plaque psoriasis, patients with mild or mild-to-moderate psoriasis and animal/in vitro studies will be excluded.

Interventions

Licensed doses of the following therapies will be included:

- Brodalumab
- Adalimumab
- Apremilast
- Dimethyl Fumarate
- Etanercept
- Infliximab
- Ixekizumab
- Secukinumab
- Ustekinumab
- Guselkumab
- Risankizumab (phase III trial dose)
- Tildrakizumab
- Certolizumab pegol

All other therapies will be excluded, including, but not limited to:

o Non-biologic systemics not listed above (e.g. methotrexate as monotherapy or in combination with biologics)

o Phototherapy

o Alternative medicine (such as homeopathy, naturopathy, and Reiki)

Comparators

Comparators for inclusion will be:

o All included interventions, including unlicensed doses of biologics and non-biologic systemics

o Placebo

Main outcomes

PASI 50, 75, 90, and 100

Additional outcome(s)

None

Study selection

The results of the electronic search will be downloaded into an EndNote library. After duplicates are removed, titles and abstracts will be assessed by one reviewer against the inclusion/exclusion criteria. 40% of the tiles and abstracts will be checked by another reviewer. Full text copies of potentially relevant articles will be retrieved in full text and further assessed against inclusion/exclusion criteria by two independent reviewers. At both stages, disagreements will be resolved by discussion or, if needed, a third reviewer.

Data extraction

For each included study, details of trial design, inclusion criteria, study population characteristics, interventions, outcome measures, results and length of follow-up will be extracted.

Risk of bias (quality) assessment

The methodological quality of included studies will be assessed by one independent reviewer and checked by another using the concise critical appraisal checklists in the National Institute for Health and Care Excellence (NICE) Single Technology Appraisal user guide.

Strategy for data synthesis

If sufficiently low heterogeneity is detected across trials, a Bayesian NMA will be performed. A random-effects multinomial likelihood model with probit link will be used and meta-regression to adjust for cross-trial variation in placebo responses performed to compare the efficacy of interventions at inducing different levels of PASI response.

Two sensitivity analyses will be performed:

1. excluding trials reporting <5% biologic exposed patients, using a different timepoint (12 weeks rather than 16 weeks) for secukinumab
2. excluding trials with fewer than 50 patients per treatment arm

Analysis of subgroups or subsets

None planned.

Funding sources/sponsors

This study is funded by Leo Pharma A/S.

Conflicts of interest

Laura Sawyer, Kinga Malottki, Najeeda Yasmeen, Emily Wright and Celia Sabry-Grant are Symmetron employees and consultants to LEO Pharma for this study; Richard Warren is a consultant to LEO Pharma for this study; Anne Sohrt and Emma Borg are LEO Pharma employees.
