# Supplementary material for: Assessing the relative efficacy of interleukin-17 and interleukin-23 targeted treatments for moderate-to-severe plaque psoriasis: A systematic review and network meta-analysis of PASI response
Source: PLoS One. 2019 Aug 14;14(8):e0220868. doi: 10.1371/journal.pone.0220868 (PMC6693782; doi:10.1371/journal.pone.0220868)
Supplement: S2 Text — (DOCX) [file pone.0220868.s002.docx]

**S2 Text. Model**

Each trial reported the number of patients in mutually exclusive categories, representing the percentage improvement in symptoms. These categories define 5 cut-off points of %PASI improvement, as follows

C=1: 0

C=2: 50

C=3: 75

C=4: 90

C=5: 100

Data are transformed to conditional binomials.

# Binomial likelihood, probit link (different categories)

# Random effects model for multi-arm trials

model{ # *** PROGRAM STARTS

for(i in 1:ns){ # LOOP THROUGH STUDIES

w[i,1] <- 0 # adjustment for multi-arm trials is zero for control arm

delta[i,1] <- 0 # treatment effect is zero for control arm

# vague priors for all trial baselines

mu[i] ~ dnorm(0,0.01)

for (k in 1:na[i]) { # LOOP THROUGH ARMS

p[i,k,1] <- 1 # Pr(PASI >0)

for (j in 1:nc[i]-1) { # LOOP THROUGH CATEGORIES

# binomial likelihood

r[i,k,j] ~ dbin(q[i,k,j],n[i,k,j])

# conditional probabilities

q[i,k,j] <- 1-(p[i,k,C[i,j+1]]/p[i,k,C[i,j]])

theta[i,k,j] <- mu[i] + delta[i,k] + (beta[t[i,k]] - beta[t[i,1]]) * (mu[i]-mx) + z[C[i,j+1]-1] # linear predictor

rhat[i,k,j] <- q[i,k,j] * n[i,k,j] # predicted number events

#Deviance contribution of each category

dv[i,k,j] <- 2 * (r[i,k,j]*(log(r[i,k,j])-log(rhat[i,k,j]))

+(n[i,k,j]-r[i,k,j])*(log(n[i,k,j]-r[i,k,j]) - log(n[i,k,j]-rhat[i,k,j])))

}

dev[i,k] <- sum(dv[i,k,1:nc[i]-1]) # deviance contribution of each arm

for (j in 2:nc[i]) { # LOOP THROUGH CATEGORIES

p[i,k,C[i,j]] <- 1 - phi.adj[i,k,j] # link function

# adjust link function phi(x) for extreme values that can give numerical errors

# when x< -5, phi(x)=0, when x> 5, phi(x)=1

phi.adj[i,k,j] <- step(5+theta[i,k,j-1])

* (step(theta[i,k,j-1]-5)

+ step(5-theta[i,k,j-1])*phi(theta[i,k,j-1]) )

}

}

for (k in 2:na[i]) { # LOOP THROUGH ARMS

delta[i,k] ~ dnorm(md[i,k],taud[i,k])

# mean of LHR distributions, with multi-arm trial correction

md[i,k] <- d[t[i,k]] - d[t[i,1]] + sw[i,k]

# precision of LHR distributions (with multi-arm trial correction)

taud[i,k] <- tau *2*(k-1)/k

# adjustment, multi-arm RCTs

w[i,k] <- (delta[i,k] - d[t[i,k]] + d[t[i,1]])

# cumulative adjustment for multi-arm trials

sw[i,k] <- sum(w[i,1:k-1])/(k-1)

}

# summed residual deviance contribution for this trial

resdev[i] <- sum(dev[i,1:na[i]])

}

z[1] <- 0 # set z50=0

#Set priors for z, for any number of categories

for (j in 2:Cmax-1) {

z.aux[j] ~ dunif(0,2) #priors

z[j] <- z[j-1] + z.aux[j] #ensures z[j]~Uniform(z[j-1],z[j-1]+5

}

totresdev <- sum(resdev[]) #Total Residual Deviance

d[1] <- 0 # treatment effect is zero for reference treatment

beta[1] <- 0 #covariate effect is zero for placebo

for (k in 2:nt){

d[k] ~ dnorm(0,0.01) # vague priors for treatment effects

beta[k] <- B

}

B ~ dnorm(0,0.01)

sd ~ dunif(0,2) # vague prior for between-trial SD

tau <- pow(sd,-2) # between-trial precision = (1/between-trial variance)
