# Supplementary material for: Assessing the relative efficacy of interleukin-17 and interleukin-23 targeted treatments for moderate-to-severe plaque psoriasis: A systematic review and network meta-analysis of PASI response
Source: PLoS One. 2019 Aug 14;14(8):e0220868. doi: 10.1371/journal.pone.0220868 (PMC6693782; doi:10.1371/journal.pone.0220868)
Supplement: S2 Table — (DOCX) [file pone.0220868.s004.docx]

**S2 Table. Study eligibility criteria**

| Criterion | Inclusion/exclusion criteria |
| --- | --- |
| Patient population | INCLUDE   - Adult patients with moderate-to-severe chronic plaque-type psoriasis   EXCLUDE   - Paediatric patients - Patients with non-plaque psoriasis - Patients with mild or mild-to-moderate psoriasis - Animal/in vitro studies |
| Intervention | INCLUDE  Active therapies – EMA licensed doses   - Brodalumab - Secukinumab - Etanercept - Infliximab - Adalimumab - Ustekinumab - Apremilast - Ixekizumab - Dimethyl Fumarate - Guselkumab - Tildrakizumab - Certolizumab pegol   Phase III doses   - Risankizumab^ǂ^   EXCLUDE   - All other therapies, including, but not limited to:   - Non-biologic systemics (e.g. methotrexate as monotherapy or in combination with biologics   - Phototherapy   - Alternative medicine (such as homeopathy, naturopathy, and Reiki) |
| Comparator | INCLUDE   - Placebo - All monotherapies, including unlicensed doses of biologics and non-biologic systemics |
| Outcomes | INCLUDE  Efficacy (short-term)   - PASI (50, 75, 90, 100)   EXCLUDE   - Outcomes beyond randomisation stage - Outcomes for treatment arms after rescue therapy has been offered |
| Study design | INCLUDE   - RCT*   EXCLUDE   - All other types of studies (non-randomised studies, long-term extensions, editorials, case reports, reviews, etc.) |
| Limits | English language only |
| Timespan | Limited to published 2000 – current |

PASI – Psoriasis Area and Severity Index; RCT – Randomised Controlled Trial

*Relevant SRs and NMAs were ordered so included study lists could be reviewed, to identify any additional relevant publications.

^ǂ^150 mg dose of risankizumab has been licensed since we carried out the study selection
