# Supplementary material for: Assessing the relative efficacy of interleukin-17 and interleukin-23 targeted treatments for moderate-to-severe plaque psoriasis: A systematic review and network meta-analysis of PASI response
Source: PLoS One. 2019 Aug 14;14(8):e0220868. doi: 10.1371/journal.pone.0220868 (PMC6693782; doi:10.1371/journal.pone.0220868)
Supplement: S5 Table — (DOCX) [file pone.0220868.s007.docx]

**S5 Table. NICE concise critical appraisal checklist for studies included in NMA**

| **Study** | **Was the randomisation method adequate** | **Was the allocation adequately concealed?** | **Were baseline characteristics similar between treatment arms?** | **Were participants and investigators blind to exposure and comparison?** | **Were discontinuations similar between groups?** | **Unreported outcomes suspected?** | **Analysis type** | **How was missing data handled?** |
| --- | --- | --- | --- | --- | --- | --- | --- | --- |
| AMAGINE-1 | Yes | Yes | Yes | Yes | Yes | No | ITT | NRI |
| AMAGINE-2 | Yes | Yes | Yes | Yes | Yes | No | ITT | NRI |
| AMAGINE-3 | Yes | Yes | Yes | Yes | Yes | No | ITT | NRI |
| Nakagawa 2016 | Yes | Unclear | Yes | Unclear | Yes | No | ITT | NRI |
| Papp 2012 | Yes | Yes | Yes | Yes | Yes | No | ITT | NRI |
| CHAMPION | Yes | Yes | Yes | Yes | Yes | No | ITT | NRI |
| Goldminz 2015 | Yes | Unclear | Yes | Partly | Yes | No | ITT | Unclear |
| Cai et al. 2016 | Yes | Yes | Yes | Yes | Unclear | No | ITT | NRI |
| REVEAL | Yes | Yes | Yes | Yes | Yes | No | ITT | NRI |
| Asahina 2010 | Unclear | Unclear | Yes | Yes | Yes | No | Per protocol | LOCF |
| Gordon 2006 | Yes | Yes | Yes | Yes | Yes | No | Modified ITT | NRI |
| X-PLORE | Unclear | Unclear | Yes | Yes | Yes | No | ITT | NRI |
| PSOR-005 | Yes | Yes | Yes | Partly | Yes | No | ITT | LOCF |
| ESTEEM 1 | Yes | Yes | Yes | Yes | No | No | Per protocol | LOCF |
| ESTEEM 2 | Yes | Yes | Yes | Yes | No | No | Modified ITT | LOCF |
| Papp 2013 | Yes | Yes | Yes | Yes | No | No | ITT | LOCF |
| Ohtsuki 2017 | Yes | Yes | Yes | Yes | Yes | No | Modified ITT | LOCF |
| LIBERATE | Yes | Yes | Yes | Yes | Yes | No | Modified ITT | LOCF |
| Leonardi 2003 | Yes | Yes | Yes | Yes | Unclear | No | Unclear | LOCF |
| Gottlieb 2003 | Yes | Yes | Yes | Yes | No | No | ITT | LOCF |
| Papp 2005 | Yes | Yes | Yes | Yes | No | No | Modified ITT | LOCF |
| Van de Kerkhof 2008 | Yes | Yes | Yes | Yes | No | No | ITT | LOCF |
| Bagel 2012 | Yes | Yes | Yes | Yes | Yes | No | ITT | LOCF |
| Bachelez 2015 | Yes | Yes | Yes | Yes | Yes | No | ITT | NRI |
| Tyring 2006 | Yes | Yes | Yes | Yes | Yes | No | ITT | LOCF |
| PRISTINE | Yes | YEs | Yes | Unclear | Yes | No | Modified ITT | LOCF |
| M10-114 | Yes | Yes | Yes | Yes | Yes | No | ITT | NRI |
| M10-315 | Yes | Yes | Yes | Yes | No | No | ITT | NRI |
| PIECE | Yes | Yes | Yes | Partly | Yes | No | ITT | LOCF |
| VOYAGE 1 | Yes | Yes | Yes | Yes | Yes | No | Yes | NRI |
| VOYAGE 2 | Yes | Unclear | Yes | Yes | Yes | No | ITT | NRI |
| Yang 2012 | Unclear | Unclear | Yes | Unclear | Yes | No | Unclear | Unclear |
| EXPRESS | Yes | Yes | Yes | Yes | Yes | No | Per protocol | NRI |
| Chaudhari 2001 | Yes | Yes | Yes | Yes | Yes | No | ITT | Unclear |
| SPIRIT | Yes | Yes | Yes | Yes | No | No | ITT | NRI |
| EXPRESS II | Yes | Yes | Yes | Yes | No | No | ITT | NRI |
| Torii 2010 | Yes | Yes | Yes | Yes | Yes | No | ITT | NRI |
| RESTORE1 | Yes | No | Yes | No | No | No | ITT | NRI |
| UNCOVER-1 | Yes | Yes | Yes | Yes | Yes | No | ITT | NRI |
| UNCOVER-2 | Yes | Yes | Yes | Yes | Yes | No | ITT | NRI |
| UNCOVER-3 | Yes | Yes | Yes | Yes | Yes | No | ITT | NRI |
| IXORA-S | Yes | Yes | Yes | Yes | Yes | No | ITT | NRI |
| Reich 2017 | Unclear | Unclear | Unclear | No | Unclear | No | Unclear | NRI |
| FEATURE | Yes | Unclear | Yes | Yes | Yes | No | Unclear | Unclear |
| ERASURE | Yes | Yes | Yes | Yes | Yes | No | Unclear | Unclear |
| FIXTURE | Yes | Yes | Yes | Yes | No | No | Unclear | Unclear |
| JUNCTURE | Yes | Yes | Yes | Yes | Yes | No | Modified ITT | LOCF |
| SCULPTURE | Yes | Yes | Yes | Unclear | No | No | Unclear | NRI |
| CLEAR | Yes | Yes | Yes | Yes | Yes | No | Per protocol | NRI |
| PRIME | Yes | Yes | Yes | No | No | No | Unclear | NRI |
| Papp 2015 | Yes | Yes | Yes | Yes | Yes | No | FAS | LOCF |
| reSURFACE 1 | Yes | Yes | Yes | Yes | Yes | No | ITT | NRI |
| reSURFACE 2 | Yes | Yes | Yes | Yes | Yes | No | ITT | NRI |
| PEARL | Yes | Yes | Yes | Yes | Yes | No | ITT | NRI |
| PHOENIX 1 | Yes | Yes | Yes | Yes | No | No | ITT | NRI |
| PHEONIX 2 | Yes | Yes | Yes | Yes | Yes | No | ITT | NRI |
| LOTUS | Yes | Yes | Yes | Yes | Yes | No | Per protocol | NRI |
| ACCEPT | Yes | Unclear | Yes | Partly | Yes | No | Unclear | Unclear |
| Igarashi 2012 | Unclear | Unclear | Yes | Unclear | Yes | No | ITT | NRI |
| BRIDGE | Yes | Yes | Yes | Yes | Yes | No | ITT | LOCF |
| Caproni 2009 | Yes | Unclear | Yes | Unclear | Unclear | No | Unclear | Unclear |
| Gisondi 2008 | Yes | Unclear | Yes | Yes | Yes | No | ITT | Unclear |
| SIGNATURE | Unclear | Unclear | Unclear | Unclear | Unclear | No | Unclear | Unclear |
| IMMhance | Yes | Unclear | Unclear | Unclear | Unclear | No | Unclear | NRI |
| Ohtsuki 2018 | Yes | Yes | Yes | Yes | No | No | ITT | NRI |
| IMMvent | Unclear | Unclear | Unclear | Yes | Unclear | No | Unclear | Unclear |
| UltiMMa-1 | Yes | Yes | Yes | Yes | Yes | No | ITT | Unclear |
| UltiMMa-2 | Yes | Yes | Yes | Yes | Yes | No | ITT | Unclear |
| Ferris 2018 | Unclear | Unclear | Yes | Yes | Unclear | No | Unclear | Unclear |
| IXORA-P | Yes | Unclear | Yes | Yes | Yes | No | ITT | NRI |
| CIMPACT | Yes | Unclear | Yes | Yes | Yes | No | ITT | MI |
| CLARITY | Unclear | Unclear | Yes | Yes | Yes | No | Unclear | MI |
| CIMPASI-1 | Yes | Yes | Yes | Yes | Unclear | No | ITT | MI |
| CIMPASI-2 | Yes | Yes | Yes | Yes | Unclear | No | ITT | MI |
| Khattri 2018 | Unclear | Unclear | Yes | Unclear | Yes | No | Unclear | NRI |
| Reich 2012 | Yes | Unclear | Yes | Yes | No | No | ITT | NRI |

ITT, intention-to-treat; LOCF, last observation carried forward; NRI, non-responder imputation; FAS, full analysis set; MI, multiple imputation
