# Supplementary material for: Assessing the relative efficacy of interleukin-17 and interleukin-23 targeted treatments for moderate-to-severe plaque psoriasis: A systematic review and network meta-analysis of PASI response
Source: PLoS One. 2019 Aug 14;14(8):e0220868. doi: 10.1371/journal.pone.0220868 (PMC6693782; doi:10.1371/journal.pone.0220868)
Supplement: S6 Table — (DOCX) [file pone.0220868.s008.docx]

**S6 Table. risk ratios for each pairwise comparison at PASI 50, 75, 90 and 100 (placebo-adjusted analysis)**

Table 1. Risk ratios for each pairwise comparison at PASI 50 from placebo-adjusted analysis. Column- vs row-defined treatment. RR>1 indicates that the column-defining treatment is more efficacious.

| BRO |  |  |  |  |  |  |  |  |  |  |  |  |  |  |  |
| --- | --- | --- | --- | --- | --- | --- | --- | --- | --- | --- | --- | --- | --- | --- | --- |
| 1 (0.98, 1.01) | IXE |  |  |  |  |  |  |  |  |  |  |  |  |  |  |
| **1.02 (1.01, 1.06)** | **1.03 (1.01, 1.07)** | SEC |  |  |  |  |  |  |  |  |  |  |  |  |  |
| 1.01 (0.99, 1.04) | 1.01 (1, 1.05) | 0.99 (0.95, 1.01) | GUS |  |  |  |  |  |  |  |  |  |  |  |  |
| 1 (0.98, 1.02) | 1 (0.99, 1.02) | **0.98 (0.94, 0.99)** | 0.99 (0.96, 1.01) | RIS |  |  |  |  |  |  |  |  |  |  |  |
| **1.17 (1.07, 1.39)** | **1.18 (1.07, 1.4)** | **1.15 (1.06, 1.32)** | **1.16 (1.06, 1.36)** | **1.18 (1.07, 1.39)** | TIL |  |  |  |  |  |  |  |  |  |  |
| **1.12 (1.05, 1.26)** | **1.12 (1.05, 1.27)** | **1.09 (1.04, 1.2)** | **1.11 (1.04, 1.23)** | **1.12 (1.05, 1.26)** | 0.96 (0.88, 1) | UST WBD |  |  |  |  |  |  |  |  |  |
| **1.12 (1.05, 1.25)** | **1.12 (1.05, 1.26)** | **1.09 (1.03, 1.19)** | **1.1 (1.04, 1.22)** | **1.12 (1.05, 1.25)** | 0.95 (0.87, 1) | 1 (0.96, 1.04) | ADA |  |  |  |  |  |  |  |  |
| **1.16 (1.06, 1.36)** | **1.16 (1.06, 1.37)** | **1.13 (1.05, 1.29)** | **1.15 (1.06, 1.33)** | **1.16 (1.06, 1.36)** | 0.99 (0.91, 1.07) | 1.03 (0.98, 1.12) | 1.04 (0.99, 1.13) | CZP 200 |  |  |  |  |  |  |  |
| **1.12 (1.04, 1.27)** | **1.12 (1.04, 1.28)** | **1.09 (1.03, 1.21)** | **1.11 (1.04, 1.25)** | **1.12 (1.04, 1.28)** | 0.96 (0.87, 1.02) | 1 (0.95, 1.06) | 1 (0.95, 1.06) | 0.97 (0.9, 1.02) | CZP 400 |  |  |  |  |  |  |
| **1.58 (1.26, 2.22)** | **1.59 (1.26, 2.24)** | **1.54 (1.25, 2.11)** | **1.56 (1.25, 2.17)** | **1.58 (1.26, 2.23)** | **1.34 (1.16, 1.67)** | **1.41 (1.2, 1.79)** | **1.42 (1.2, 1.81)** | **1.36 (1.17, 1.7)** | **1.41 (1.2, 1.8)** | ETA 50 |  |  |  |  |  |
| **1.32 (1.14, 1.67)** | **1.33 (1.14, 1.68)** | **1.29 (1.13, 1.58)** | **1.31 (1.14, 1.63)** | **1.33 (1.14, 1.67)** | **1.12 (1.05, 1.25)** | **1.18 (1.09, 1.34)** | **1.19 (1.09, 1.36)** | **1.14 (1.06, 1.28)** | **1.18 (1.08, 1.36)** | **0.84 (0.73, 0.92)** | ETA 100 |  |  |  |  |
| **1.05 (1.02, 1.12)** | **1.05 (1.02, 1.13)** | 1.02 (1, 1.07) | **1.04 (1.01, 1.1)** | **1.05 (1.02, 1.12)** | **0.89 (0.79, 0.96)** | **0.94 (0.87, 0.98)** | **0.94 (0.88, 0.98)** | **0.91 (0.81, 0.97)** | **0.94 (0.86, 0.98)** | **0.66 (0.5, 0.81)** | **0.79 (0.66, 0.89)** | INF |  |  |  |
| **1.89 (1.4, 2.88)** | **1.9 (1.4, 2.9)** | **1.84 (1.39, 2.73)** | **1.87 (1.4, 2.82)** | **1.89 (1.4, 2.89)** | **1.6 (1.3, 2.15)** | **1.69 (1.33, 2.32)** | **1.69 (1.34, 2.33)** | **1.62 (1.31, 2.19)** | **1.68 (1.33, 2.33)** | **1.19 (1.06, 1.4)** | **1.42 (1.22, 1.78)** | **1.8 (1.38, 2.61)** | APR |  |  |
| **1.94 (1.4, 3.17)** | **1.95 (1.4, 3.2)** | **1.9 (1.38, 3.03)** | **1.92 (1.39, 3.11)** | **1.95 (1.4, 3.18)** | **1.65 (1.28, 2.41)** | **1.73 (1.32, 2.59)** | **1.74 (1.32, 2.61)** | **1.67 (1.29, 2.46)** | **1.73 (1.32, 2.6)** | **1.22 (1.01, 1.61)** | **1.46 (1.18, 2.02)** | **1.85 (1.37, 2.9)** | 1.03 (0.84, 1.31) | DMF |  |
| **6.52 (3.39, 14.63)** | **6.54 (3.39, 14.75)** | **6.37 (3.36, 13.91)** | **6.45 (3.37, 14.3)** | **6.53 (3.39, 14.68)** | **5.55 (3.15, 10.77)** | **5.82 (3.23, 11.73)** | **5.84 (3.23, 11.83)** | **5.61 (3.17, 11.02)** | **5.82 (3.23, 11.74)** | **4.11 (2.66, 6.76)** | **4.92 (2.96, 8.83)** | **6.22 (3.32, 13.23)** | **3.44 (2.38, 5.25)** | **3.32 (2.26, 5.31)** | PBO |

**Note:** Values are provided for the comparison of column-defined treatment vs row-defined treatment. RR>1 indicates that the column-defining treatment is more efficacious. Grey and bolded values indicate that differences were statistically significant.

Table 2. Risk ratios for each pairwise comparison at PASI 75 from placebo-adjusted analysis. Column- vs row-defined treatment. RR>1 indicates that the column-defining treatment is more efficacious.

| BRO |  |  |  |  |  |  |  |  |  |  |  |  |  |  |  |
| --- | --- | --- | --- | --- | --- | --- | --- | --- | --- | --- | --- | --- | --- | --- | --- |
| 0.99 (0.95, 1.03) | IXE |  |  |  |  |  |  |  |  |  |  |  |  |  |  |
| **1.06 (1.02, 1.14)** | **1.07 (1.02, 1.15)** | SEC |  |  |  |  |  |  |  |  |  |  |  |  |  |
| 1.02 (0.98, 1.09) | 1.03 (0.99, 1.1) | 0.97 (0.91, 1.02) | GUS |  |  |  |  |  |  |  |  |  |  |  |  |
| 1 (0.95, 1.04) | 1.01 (0.97, 1.05) | **0.94 (0.87, 0.98)** | 0.97 (0.91, 1.01) | RIS |  |  |  |  |  |  |  |  |  |  |  |
| **1.39 (1.18, 1.78)** | **1.4 (1.18, 1.81)** | **1.31 (1.14, 1.62)** | **1.35 (1.16, 1.71)** | **1.39 (1.18, 1.79)** | TIL |  |  |  |  |  |  |  |  |  |  |
| **1.27 (1.13, 1.52)** | **1.28 (1.13, 1.55)** | **1.2 (1.09, 1.37)** | **1.24 (1.11, 1.46)** | **1.27 (1.13, 1.53)** | 0.92 (0.8, 1.01) | UST WBD |  |  |  |  |  |  |  |  |  |
| **1.26 (1.12, 1.5)** | **1.27 (1.12, 1.53)** | **1.19 (1.09, 1.36)** | **1.23 (1.11, 1.43)** | **1.26 (1.12, 1.51)** | 0.91 (0.79, 1) | 0.99 (0.92, 1.07) | ADA |  |  |  |  |  |  |  |  |
| **1.35 (1.16, 1.72)** | **1.37 (1.16, 1.75)** | **1.28 (1.12, 1.57)** | **1.32 (1.14, 1.66)** | **1.36 (1.16, 1.74)** | 0.98 (0.85, 1.12) | 1.07 (0.97, 1.22) | 1.07 (0.98, 1.23) | CZP 200 |  |  |  |  |  |  |  |
| **1.27 (1.12, 1.55)** | **1.28 (1.12, 1.58)** | **1.19 (1.08, 1.42)** | **1.23 (1.1, 1.49)** | **1.27 (1.12, 1.56)** | 0.92 (0.78, 1.03) | 1 (0.91, 1.11) | 1.01 (0.92, 1.12) | 0.94 (0.83, 1.03) | CZP 400 |  |  |  |  |  |  |
| **2.28 (1.61, 3.59)** | **2.3 (1.62, 3.65)** | **2.15 (1.57, 3.25)** | **2.22 (1.59, 3.43)** | **2.29 (1.61, 3.62)** | **1.63 (1.32, 2.18)** | **1.79 (1.41, 2.45)** | **1.8 (1.42, 2.47)** | **1.67 (1.34, 2.24)** | **1.79 (1.41, 2.47)** | ETA 50 |  |  |  |  |  |
| **1.71 (1.35, 2.36)** | **1.73 (1.35, 2.4)** | **1.62 (1.31, 2.13)** | **1.67 (1.33, 2.26)** | **1.72 (1.35, 2.38)** | **1.23 (1.09, 1.44)** | **1.34 (1.18, 1.6)** | **1.36 (1.19, 1.63)** | **1.26 (1.11, 1.49)** | **1.34 (1.17, 1.63)** | **0.75 (0.62, 0.86)** | ETA 100 |  |  |  |  |
| **1.11 (1.04, 1.24)** | **1.12 (1.05, 1.26)** | 1.05 (1, 1.14) | **1.08 (1.02, 1.2)** | **1.12 (1.04, 1.25)** | **0.81 (0.67, 0.91)** | **0.88 (0.78, 0.95)** | **0.89 (0.79, 0.95)** | **0.82 (0.69, 0.92)** | **0.88 (0.76, 0.96)** | **0.49 (0.33, 0.66)** | **0.65 (0.51, 0.78)** | INF |  |  |  |
| **2.98 (1.94, 5.16)** | **3.01 (1.95, 5.25)** | **2.82 (1.88, 4.68)** | **2.91 (1.91, 4.95)** | **2.99 (1.94, 5.21)** | **2.14 (1.6, 3.1)** | **2.35 (1.7, 3.5)** | **2.36 (1.71, 3.54)** | **2.19 (1.63, 3.2)** | **2.34 (1.7, 3.53)** | **1.3 (1.09, 1.63)** | **1.74 (1.4, 2.29)** | **2.67 (1.83, 4.29)** | APR |  |  |
| **3.11 (1.92, 5.95)** | **3.14 (1.93, 6.04)** | **2.94 (1.86, 5.4)** | **3.03 (1.9, 5.7)** | **3.12 (1.92, 5.98)** | **2.22 (1.55, 3.66)** | **2.44 (1.67, 4.11)** | **2.46 (1.67, 4.16)** | **2.28 (1.58, 3.78)** | **2.44 (1.66, 4.14)** | **1.36 (1.02, 1.99)** | **1.81 (1.33, 2.78)** | **2.79 (1.8, 5)** | 1.04 (0.77, 1.47) | DMF |  |
| **16.26 (7.07, 42.54)** | **16.42 (7.1, 43.31)** | **15.36 (6.89, 38.4)** | **15.85 (6.99, 40.62)** | **16.32 (7.08, 42.86)** | **11.67 (5.9, 24.89)** | **12.8 (6.25, 28.47)** | **12.88 (6.27, 28.87)** | **11.96 (6, 25.72)** | **12.8 (6.24, 28.54)** | **7.1 (4.3, 12.37)** | **9.5 (5.22, 18.28)** | **14.58 (6.71, 35.1)** | **5.42 (3.54, 8.7)** | **5.14 (3.22, 8.96)** | PBO |

**Note:** Values are provided for the comparison of column-defined treatment vs row-defined treatment. RR>1 indicates that the column-defining treatment is more efficacious. Grey and bolded values indicate that differences were statistically significant.

Table 3. Risk ratios for each pairwise comparison at PASI 90 from placebo-adjusted analysis. Column- vs row-defined treatment. RR>1 indicates that the column-defining treatment is more efficacious.

| BRO |  |  |  |  |  |  |  |  |  |  |  |  |  |  |  |
| --- | --- | --- | --- | --- | --- | --- | --- | --- | --- | --- | --- | --- | --- | --- | --- |
| 0.98 (0.9, 1.06) | IXE |  |  |  |  |  |  |  |  |  |  |  |  |  |  |
| **1.13 (1.04, 1.28)** | **1.15 (1.06, 1.32)** | SEC |  |  |  |  |  |  |  |  |  |  |  |  |  |
| 1.05 (0.96, 1.19) | 1.08 (0.99, 1.22) | 0.94 (0.83, 1.04) | GUS |  |  |  |  |  |  |  |  |  |  |  |  |
| 0.99 (0.9, 1.09) | 1.01 (0.93, 1.11) | **0.88 (0.77, 0.96)** | 0.94 (0.83, 1.03) | RIS |  |  |  |  |  |  |  |  |  |  |  |
| **1.88 (1.45, 2.67)** | **1.92 (1.47, 2.75)** | **1.66 (1.33, 2.24)** | **1.77 (1.39, 2.47)** | **1.89 (1.45, 2.71)** | TIL |  |  |  |  |  |  |  |  |  |  |
| **1.6 (1.32, 2.07)** | **1.64 (1.34, 2.15)** | **1.42 (1.22, 1.72)** | **1.51 (1.27, 1.93)** | **1.62 (1.33, 2.11)** | 0.86 (0.69, 1.02) | UST WBD |  |  |  |  |  |  |  |  |  |
| **1.58 (1.31, 2.03)** | **1.62 (1.32, 2.11)** | **1.4 (1.2, 1.71)** | **1.5 (1.27, 1.87)** | **1.6 (1.32, 2.06)** | 0.84 (0.68, 1) | 0.99 (0.87, 1.12) | ADA |  |  |  |  |  |  |  |  |
| **1.8 (1.4, 2.54)** | **1.84 (1.42, 2.63)** | **1.59 (1.29, 2.14)** | **1.7 (1.35, 2.37)** | **1.82 (1.41, 2.59)** | 0.96 (0.76, 1.21) | 1.12 (0.94, 1.39) | 1.14 (0.96, 1.41) | CZP 200 |  |  |  |  |  |  |  |
| **1.6 (1.29, 2.15)** | **1.63 (1.31, 2.23)** | **1.41 (1.18, 1.82)** | **1.51 (1.24, 2.01)** | **1.61 (1.3, 2.19)** | 0.85 (0.67, 1.06) | 1 (0.84, 1.2) | 1.01 (0.86, 1.21) | 0.89 (0.73, 1.05) | CZP 400 |  |  |  |  |  |  |
| **4.17 (2.57, 7.42)** | **4.26 (2.6, 7.67)** | **3.68 (2.38, 6.18)** | **3.93 (2.48, 6.84)** | **4.2 (2.58, 7.55)** | **2.2 (1.63, 3.21)** | **2.58 (1.88, 3.83)** | **2.62 (1.9, 3.9)** | **2.29 (1.69, 3.37)** | **2.58 (1.85, 3.93)** | ETA 50 |  |  |  |  |  |
| **2.66 (1.88, 4.05)** | **2.72 (1.91, 4.18)** | **2.35 (1.75, 3.35)** | **2.52 (1.82, 3.74)** | **2.69 (1.89, 4.13)** | **1.41 (1.17, 1.76)** | **1.65 (1.37, 2.09)** | **1.68 (1.39, 2.13)** | **1.47 (1.21, 1.87)** | **1.65 (1.34, 2.16)** | **0.64 (0.5, 0.78)** | ETA 100 |  |  |  |  |
| **1.25 (1.11, 1.5)** | **1.28 (1.13, 1.54)** | 1.11 (1, 1.27) | **1.18 (1.05, 1.4)** | **1.26 (1.11, 1.52)** | **0.67 (0.51, 0.82)** | **0.78 (0.65, 0.9)** | **0.8 (0.66, 0.91)** | **0.7 (0.53, 0.85)** | **0.79 (0.62, 0.93)** | **0.3 (0.19, 0.45)** | **0.47 (0.34, 0.61)** | INF |  |  |  |
| **6.22 (3.48, 12.28)** | **6.35 (3.52, 12.7)** | **5.49 (3.22, 10.22)** | **5.87 (3.36, 11.33)** | **6.27 (3.5, 12.46)** | **3.28 (2.24, 5.22)** | **3.86 (2.55, 6.29)** | **3.91 (2.58, 6.38)** | **3.42 (2.32, 5.47)** | **3.85 (2.54, 6.42)** | **1.48 (1.14, 2.02)** | **2.32 (1.75, 3.29)** | **4.94 (3.01, 8.8)** | APR |  |  |
| **6.6 (3.4, 15.09)** | **6.75 (3.44, 15.57)** | **5.82 (3.13, 12.63)** | **6.24 (3.28, 13.83)** | **6.66 (3.41, 15.15)** | **3.48 (2.11, 6.66)** | **4.09 (2.43, 7.95)** | **4.15 (2.46, 8.1)** | **3.62 (2.19, 6.95)** | **4.08 (2.41, 8.06)** | **1.57 (1.02, 2.67)** | **2.46 (1.6, 4.34)** | **5.24 (2.9, 11.05)** | 1.06 (0.68, 1.73) | DMF |  |
| **62.34 (22.61, 188.04)** | **63.73 (22.91, 194.67)** | **55.07 (21.08, 155.69)** | **58.89 (21.9, 173.16)** | **62.89 (22.72, 191.53)** | **32.96 (15.02, 76.8)** | **38.74 (16.84, 94.52)** | **39.22 (16.98, 96.43)** | **34.34 (15.43, 80.91)** | **38.74 (16.76, 95.33)** | **14.86 (8.45, 27.43)** | **23.35 (11.87, 47.69)** | **49.6 (19.72, 133.53)** | **9.96 (6.16, 16.86)** | **9.27 (5.17, 18.03)** | PBO |

**Note:** Values are provided for the comparison of column-defined treatment vs row-defined treatment. RR>1 indicates that the column-defining treatment is more efficacious. Grey and bolded values indicate that differences were statistically significant.

Table 4. Risk ratios for each pairwise comparison at PASI 100 from placebo-adjusted analysis. Column- vs row-defined treatment. RR>1 indicates that the column-defining treatment is more efficacious.

| BRO |  |  |  |  |  |  |  |  |  |  |  |  |  |  |  |
| --- | --- | --- | --- | --- | --- | --- | --- | --- | --- | --- | --- | --- | --- | --- | --- |
| 0.96 (0.81, 1.12) | IXE |  |  |  |  |  |  |  |  |  |  |  |  |  |  |
| **1.26 (1.08, 1.55)** | **1.32 (1.13, 1.63)** | SEC |  |  |  |  |  |  |  |  |  |  |  |  |  |
| 1.11 (0.93, 1.37) | 1.16 (0.97, 1.44) | 0.88 (0.72, 1.07) | GUS |  |  |  |  |  |  |  |  |  |  |  |  |
| 0.98 (0.82, 1.17) | 1.03 (0.86, 1.23) | **0.78 (0.62, 0.93)** | 0.88 (0.71, 1.05) | RIS |  |  |  |  |  |  |  |  |  |  |  |
| **3.03 (2.08, 4.86)** | **3.18 (2.16, 5.12)** | **2.39 (1.73, 3.6)** | **2.72 (1.89, 4.27)** | **3.09 (2.1, 4.99)** | TIL |  |  |  |  |  |  |  |  |  |  |
| **2.35 (1.78, 3.29)** | **2.45 (1.83, 3.51)** | **1.85 (1.49, 2.41)** | **2.1 (1.6, 2.94)** | **2.39 (1.79, 3.42)** | 0.77 (0.56, 1.03) | UST WBD |  |  |  |  |  |  |  |  |  |
| **2.29 (1.74, 3.22)** | **2.39 (1.79, 3.41)** | **1.8 (1.44, 2.39)** | **2.05 (1.6, 2.78)** | **2.33 (1.77, 3.28)** | 0.76 (0.55, 1) | 0.98 (0.79, 1.21) | ADA |  |  |  |  |  |  |  |  |
| **2.84 (1.96, 4.52)** | **2.97 (2.03, 4.8)** | **2.24 (1.62, 3.38)** | **2.55 (1.79, 4.01)** | **2.89 (1.98, 4.66)** | 0.94 (0.65, 1.35) | 1.21 (0.9, 1.68) | 1.24 (0.93, 1.71) | CZP 200 |  |  |  |  |  |  |  |
| **2.33 (1.69, 3.53)** | **2.43 (1.74, 3.75)** | **1.83 (1.38, 2.65)** | **2.09 (1.53, 3.13)** | **2.37 (1.7, 3.63)** | 0.77 (0.53, 1.1) | 1 (0.75, 1.35) | 1.02 (0.78, 1.37) | 0.82 (0.61, 1.09) | CZP 400 |  |  |  |  |  |  |
| **10.24 (5.53, 20.57)** | **10.72 (5.72, 21.73)** | **8.06 (4.66, 15.08)** | **9.17 (5.11, 17.91)** | **10.43 (5.61, 21.17)** | **3.34 (2.21, 5.44)** | **4.33 (2.88, 7.09)** | **4.44 (2.93, 7.27)** | **3.56 (2.33, 5.88)** | **4.34 (2.79, 7.42)** | ETA 50 |  |  |  |  |  |
| **5.26 (3.35, 8.76)** | **5.5 (3.46, 9.23)** | **4.14 (2.82, 6.37)** | **4.7 (3.07, 7.7)** | **5.35 (3.37, 9.07)** | **1.72 (1.29, 2.36)** | **2.23 (1.73, 3.02)** | **2.28 (1.76, 3.11)** | **1.83 (1.35, 2.58)** | **2.23 (1.64, 3.21)** | **0.52 (0.37, 0.68)** | ETA 100 |  |  |  |  |
| **1.53 (1.24, 2.01)** | **1.6 (1.3, 2.1)** | 1.21 (1, 1.51) | **1.37 (1.1, 1.79)** | **1.56 (1.25, 2.07)** | **0.51 (0.34, 0.69)** | **0.66 (0.5, 0.83)** | **0.67 (0.51, 0.84)** | **0.54 (0.37, 0.74)** | **0.66 (0.46, 0.87)** | **0.15 (0.08, 0.25)** | **0.29 (0.2, 0.41)** | INF |  |  |  |
| **18.2 (8.81, 40.68)** | **19.01 (9.14, 43.14)** | **14.31 (7.42, 29.78)** | **16.3 (8.16, 35.31)** | **18.53 (8.94, 41.65)** | **5.93 (3.62, 10.53)** | **7.71 (4.62, 13.81)** | **7.89 (4.75, 14.11)** | **6.32 (3.85, 11.29)** | **7.69 (4.55, 14.38)** | **1.76 (1.22, 2.68)** | **3.44 (2.39, 5.29)** | **11.8 (6.41, 23.5)** | APR |  |  |
| **19.75 (8.38, 54.62)** | **20.67 (8.64, 57.81)** | **15.55 (6.99, 40.34)** | **17.72 (7.72, 47.39)** | **20.14 (8.49, 55.51)** | **6.44 (3.26, 14.84)** | **8.38 (4.21, 19.28)** | **8.57 (4.31, 19.86)** | **6.87 (3.48, 15.82)** | **8.38 (4.13, 19.89)** | **1.92 (1.04, 3.94)** | **3.74 (2.04, 7.84)** | **12.82 (5.95, 32.47)** | 1.09 (0.59, 2.14) | DMF |  |
| **398.09 (124.07, 1348.07)** | **415.97 (127.94, 1436.99)** | **313.68 (104.99, 976.56)** | **356 (114.8, 1173.71)** | **405.52 (125.87, 1392.95)** | **129.52 (54, 327.55)** | **168.83 (66.89, 444.05)** | **172.5 (67.93, 456.2)** | **138.72 (56.63, 355.49)** | **169.03 (66.05, 455.17)** | **38.52 (20.58, 75.53)** | **75.41 (36.11, 161.84)** | **258.53 (91.49, 765.11)** | **21.66 (12.65, 39.09)** | **19.69 (9.46, 43.84)** | PBO |

**Note:** Values are provided for the comparison of column-defined treatment vs row-defined treatment. RR>1 indicates that the column-defining treatment is more efficacious. Grey and bolded values indicate that differences were statistically significant.
