# Supplementary material for: Assessing the relative efficacy of interleukin-17 and interleukin-23 targeted treatments for moderate-to-severe plaque psoriasis: A systematic review and network meta-analysis of PASI response
Source: PLoS One. 2019 Aug 14;14(8):e0220868. doi: 10.1371/journal.pone.0220868 (PMC6693782; doi:10.1371/journal.pone.0220868)
Supplement: S7 Table — (DOCX) [file pone.0220868.s009.docx]

**S7 Table. Results of NMA without placebo adjustment**

Treatment effects at each level of PASI response for interventions versus base case analysis without placebo arm adjustment

| **Class** | **Treatment** | **Risk ratio versus placebo, median (95% CrI)** | | | |
| --- | --- | --- | --- | --- | --- |
|  |  | **PASI 50** | **PASI 75** | **PASI 90** | **PASI 100** |
| IL-17RA | Brodalumab 210mg | 6.50 (3.40 to 14.55) | 16.05 (7.10 to 41.65) | 60.83 (22.54 to 181.19) | 380.95 (121.55 to 1278.61) |
| IL-17A | Ixekizumab 80mg Q2W | 6.57 (3.42 to 14.94) | 16.50 (7.19 to 43.96) | 64.81 (23.41 to 201.09) | 432.15 (133.24 to 1532.57) |
|  | Secukinumab 300mg | 6.39 (3.38 to 13.98) | 15.4 (6.95 to 38.7) | 55.59 (21.38 to 158.35) | 319.69 (108.19 to 1010.71) |
| IL-23 | Guselkumab 100 mg | 6.38 (3.38 to 13.96) | 15.34 (6.95 to 38.61) | 55.19 (21.30 to 158.23) | 316.06 (106.73 to 1013.17) |
|  | Risankizumab 150 mg | 6.49 (3.40 to 14.45) | 15.93 (7.07 to 41.22) | 59.88 (22.37 to 177.97) | 369.28 (118.96 to 1231.22) |
|  | Tildrakizumab 100 mg | 5.61 (3.19 to 11.06) | 11.87 (6.02 to 25.83) | 34.00 (15.43 to 81.70) | 137.02 (55.90 to 362.19) |
| IL- 12/23 | Ustekinumab (in-label dose) | 5.78 (3.23 to 11.56) | 12.57 (6.22 to 27.75) | 37.55 (16.63 to 90.42) | 160.72 (65.15 to 420.17) |
| Anti-TNF | Adalimumab 40mg Q2W | 5.7 (3.21 to 11.31) | 12.24 (6.13 to 26.78) | 35.95 (16.13 to 85.69) | 149.52 (61.27 to 383.29) |
|  | Certolizumab 200mg | 5.41 (3.13 to 10.48) | 11.15 (5.78 to 23.75) | 30.56 (14.31 to 71.99) | 115.41 (48.73 to 301.84) |
|  | Certolizumab 400mg | 5.65 (3.20 to 11.25) | 12.06 (6.06 to 26.68) | 35.01 (15.74 to 85.62) | 143.78 (58.14 to 390.78) |
|  | Etanercept 50 mg / week | 4.07 (2.65 to 6.70) | 6.99 (4.25 to 12.24) | 14.52 (8.26 to 27.11) | 37.29 (19.83 to 74.74) |
|  | Etanercept 100 mg / week | 4.98 (3.00 to 9.03) | 9.65 (5.31 to 18.81) | 24.03 (12.22 to 49.93) | 79.05 (37.71 to 173.40) |
|  | Infliximab 5mg/kg | 6.23 (3.35 to 13.32) | 14.60 (6.76 to 35.39) | 49.80 (19.96 to 135.98) | 261.57 (92.42 to 792.39) |
| PDE4 | Apremilast 30mg BID | 3.13 (2.23 to 4.67) | 4.71 (3.18 to 7.41) | 8.14 (5.17 to 13.65) | 16.38 (9.62 to 29.65) |
| FAE | Dimethyl Fumarate | 2.52 (1.77 to 3.92) | 3.48 (2.19 to 5.98) | 5.34 (2.89 to 10.53) | 9.19 (4.10 to 21.68) |

BID, twice daily; PASI, Psoriasis Area and Severity Index; mg, milligram; kg, kilogram; Q2W, every 2 weeks; Q4W, every 4 weeks.
