# Supplementary material for: Assessing the relative efficacy of interleukin-17 and interleukin-23 targeted treatments for moderate-to-severe plaque psoriasis: A systematic review and network meta-analysis of PASI response
Source: PLoS One. 2019 Aug 14;14(8):e0220868. doi: 10.1371/journal.pone.0220868 (PMC6693782; doi:10.1371/journal.pone.0220868)
Supplement: S8 Table — (DOCX) [file pone.0220868.s010.docx]

**S8 Table. Results of sensitivity analysis removing studies reporting <5% of patients with prior biologic exposure**

Treatment effects at each level of PASI response for interventions versus placebo – placebo-adjusted SA excluding studies of fewer than 5% of patients with prior biologic exposure and where prior biologic exposure was not reported.

| **Treatment** | **Risk ratio versus placebo, median (95% Credible Interval)** | | | |
| --- | --- | --- | --- | --- |
|  | **PASI 50** | **PASI 75** | **PASI 90** | **PASI 100** |
| Brodalumab 210mg | 7.23  (4.13 to 13.93) | 16.88  (8.46 to 36.55) | 64.94  (28.22 to 158) | 412.54  (158.93 to 1108.4) |
| Ixekizumab 80mg Q2W | 7.28  (4.14 to 14.13) | 17.11  (8.53 to 37.51) | 67.02  (28.82 to 166.09) | 439.37  (166.5 to 1206.56) |
| Secukinumab 300mg | 7.02  (4.07 to 13.22) | 15.85  (8.17 to 33.06) | 56.69  (25.89 to 131.2) | 318.27  (131.56 to 803.21) |
| Guselkumab 100 mg | 7.14  (4.11 to 13.61) | 16.42  (8.33 to 34.87) | 61.05  (27.16 to 145.22) | 365.63  (145.45 to 957.85) |
| Risankizumab 150 mg | 7.27  (4.14 to 14.1) | 17.08  (8.52 to 37.34) | 66.71  (28.71 to 164.77) | 435.54  (165.29 to 1205.69) |
| Tildrakizumab 100 mg | 6.05  (3.74 to 10.42) | 12.03  (6.85 to 22.31) | 34.01  (17.78 to 68.21) | 133.44  (64.43 to 289.44) |
| Ustekinumab (in-label dose) | 6.38  (3.87 to 11.26) | 13.21  (7.3 to 25.21) | 40.05  (20.24 to 82.37) | 173.79  (81.63 to 382.7) |
| Adalimumab 40mg Q2W | 6.41  (3.88 to 11.37) | 13.36  (7.36 to 25.65) | 40.87  (20.5 to 85.03) | 179.66  (83.19 to 403.23) |
| Certolizumab 200mg | 6.11  (3.77 to 10.59) | 12.26  (6.93 to 22.9) | 35.16  (18.18 to 71.23) | 140.71  (66.89 to 310.08) |
| Certolizumab 400mg | 6.37  (3.86 to 11.24) | 13.17  (7.27 to 25.21) | 39.84  (19.99 to 83.33) | 172.65  (79.24 to 394.94) |
| Etanercept 50 mg / week | 5.31  (3.46 to 8.53) | 9.71  (5.94 to 16.49) | 23.86  (13.72 to 42.72) | 76.69  (41.77 to 143.80) |
| Infliximab 5mg/kg | 6.85  (4.03 to 12.75) | 15.14  (7.96 to 30.87) | 51.71  (24.21 to 117.1) | 269.69  (113.92 to 674.31) |
| Apremilast 30mg BID | 3.78  (2.71 to 5.53) | 5.85  (3.97 to 9.05) | 11.05  (6.97 to 18.43) | 24.91  (14.28 to 45.62) |

BID, twice daily; PASI, Psoriasis Area and Severity Index; mg, milligram; kg, kilogram; Q2W, every 2 weeks; Q4W, every 4 weeks.
